# Incidence of and mortality risk from cardiovascular diseases after a cancer diagnosis: An umbrella review

**DOI:** 10.1007/s10238-026-02295-0

**Published:** 2026-08-17

**Authors:** Sewunet Admasu Belachew, Solomon Ahmed Mohammed, Gail Garvey

**Affiliations:** 1 First Nations Cancer and Wellbeing Research Program, School of Public Health, The University of Queensland, 266 Herston Rd, Brisbane, QLD 4006 Australia; 2 Frazer Institute, Faculty of Health, Medicine and Behavioural Sciences, The University of Queensland, Brisbane, Australia

**Keywords:** Cancer, Cardiovascular disease, CVD, Incidence, Mortality, Risk

## Abstract

Cardiovascular disease (CVD) is a leading cause of morbidity and mortality among cancer survivors, driven by the cancer itself, its treatments, and shared risk factors. Despite numerous systematic reviews and meta-analyses, the evidence remains fragmented and inconsistent, limiting its clinical utility. This umbrella review synthesised evidence on the incidence of CVD and CVD-related mortality after a cancer diagnosis and critically appraised the methodological quality of the literature. A comprehensive search of PubMed, Embase, CINAHL, Web of Science, and the Cochrane Library from inception to February 2025 identified 34 eligible reviews from 12,942 records. Cancer patients consistently showed higher risks of CVD (relative risks, 1.11–2.32) and CVD-related mortality (hazard ratios, 1.09–1.70) compared with cancer-free populations, with variation by cancer type and CVD subtype. Reported outcomes included stroke, atrial fibrillation, and thromboembolic events, with evidence also indicating that patients with pre-existing CVD may develop additional cardiovascular events after cancer diagnosis. The methodological quality of included reviews ranged from critically low to moderately high, with none rated as high quality. These findings confirm a substantial cardiovascular burden among cancer patients while highlighting important limitations in the evidence base. Strengthening integrated cardio-oncology care and prioritising high-quality research are essential to improve risk stratification and patient outcomes.

**Supplementary Information:**

The online version contains supplementary material available at https://doi.org/10.1007/s10238-026-02295-0.

## Introduction

Cancer remains a major global public health challenge and a leading contributor to the worldwide disease burden [1]. In 2022, an estimated 20 million new cancer cases and 9.7 million deaths were reported globally [2]. Advances in screening, early detection, and treatment have improved cancer survival [3]. However, many survivors experience long-term health complications and increased mortality from non-cancer causes [4]. Among these, cardiovascular disease (CVD) has emerged as a major contributor to morbidity and mortality in cancer survivors [5].

The increased risk of CVD among cancer survivors arises from multiple factors, including the cancer itself [6], cancer treatments [7, 8], and patient-related factors [9, 10]. Treatments such as chemotherapy and radiotherapy can damage the heart and vasculature, leading to conditions ranging from subclinical cardiac dysfunction to severe outcomes such as hypertension, arrhythmias, myocardial infarction (MI), thromboembolism, and heart failure [7, 8]. Cancer and CVD also share common risk factors, including older age, smoking, obesity, and lack of physical activity [9, 10]. In addition, shared biological mechanisms, such as chronic inflammation, oxidative stress, metabolic dysregulation, and genetic susceptibility, further link these conditions [6, 10–12].

Several systematic reviews and meta-analyses have reported the incidence of and risk of mortality from CVD after a cancer diagnosis [13–26]. However, important clinical and methodological uncertainties remain. Existing reviews have focused on specific cancer types, individual cardiovascular outcomes, or selected patient populations, resulting in fragmented evidence. Furthermore, findings across reviews are often inconsistent because of differences in outcome definitions, follow-up periods, and CVD risk estimates. Therefore, a robust assessment of their methodological quality and a comprehensive synthesis of the evidence is needed. Umbrella reviews comprehensively summarise evidence from multiple systematic reviews and meta-analyses on the same topic by assessing methodological rigour.

This umbrella review aimed to systematically identify and synthesise evidence from published systematic reviews and meta-analyses examining the incidence of CVD and the risk of CVD-related mortality among individuals after a cancer diagnosis, and to assess the methodological quality of the evidence to provide a comprehensive understanding of the CVD burden in this population.

## Methods

The protocol for this review was registered at the International Prospective Register of Systematic Reviews (PROSPERO; registration number: CRD420261294517). The umbrella review followed the Preferred Reporting Items for Systematic Reviews and Meta-Analyses reporting guideline [27] and the Meta-analysis of Observational Studies in Epidemiology reporting guideline [28].

### Literature search and selection criteria

A comprehensive literature search was conducted in PubMed, Embase, CINAHL, Web of Science, and the Cochrane Library from database inception until February 2025 to identify systematic reviews and/or meta-analyses reporting the incidence of CVD and/or CVD-related mortality after a cancer diagnosis. Reference lists of included reviews were also screened for additional relevant studies. Our search strategy combined terms associated with cancer, CVD, risk (e.g., incidence, survival, mortality), and review type (e.g., systematic review, meta-analysis) (Supplemental Table 1). Two reviewers (SAM and MHJ) independently screened titles, abstracts, and full texts, with disagreements resolved through discussion with a third reviewer (SAB).

We included systematic reviews and/or meta-analyses that met the following criteria: (1) the study populations included individuals diagnosed with cancer, regardless of race, age, or disease stage; (2) cancer was the exposure of interest, with the general population or cancer-free individuals as comparators, or with no comparator group; and (3) the outcomes of interest included the incidence of CVD and/or CVD-related mortality after a cancer diagnosis. Reviews including patients with pre-existing CVD were eligible if they reported new cardiovascular events occurring after cancer diagnosis. New CVD was defined as incident cardiovascular events during follow-up after cancer diagnosis, despite baseline CVD. When reviews contained overlapping primary studies, the review including all studies from the other review(s) plus additional studies was selected. If the reviews included the same studies, the most recently published review was chosen.

Given that we aimed to synthesise the broader evidence of CVD-related outcomes rather than cancer treatment-specific toxicities, systematic reviews or meta-analyses focusing solely on the adverse effects of a specific cancer treatment were excluded. Likewise, reviews that did not specify the timing of CVD diagnosis or that did not clearly distinguish new-onset from pre-existing CVD were also excluded. Reviews that did not present a systematic search strategy, with clearly defined inclusion and exclusion criteria, as well as protocols, animal studies, abstracts, conference proceedings, and editorial letters were also excluded.

As defined by the International Cardio-Oncology Society, CVD is a group of disorders of the heart and blood vessels, including cardiac dysfunction, myocarditis, hypertension, arrhythmias, and vascular toxicities [29]. Vascular toxicity includes arterial thromboembolism, arterial injury, cerebrovascular ischemia, myocardial infarction, peripheral ischemia, thromboembolic event, transient ischemic attack, and vasculitis. These outcomes were considered CVD outcomes a priori. Given the heterogeneity of CVD outcome endpoints and follow-up periods across the included reviews, CVD outcomes and follow-up durations were reported as defined by the reviews.

### Data extraction and quality assessment

Two reviewers (SAB and SAM) independently extracted data, with any discrepancies referred to a third reviewer (GG) for resolution. We extracted data at both the systematic review and meta-analysis and individual study levels.

The same reviewers used the Assessment of Multiple Systematic Reviews, version 2 (AMSTAR-2) tool to grade the methodological quality of each systematic review and meta-analysis, which rated quality as critically low, low, moderate, or high [30]. Discrepancies were resolved through discussions.

### Data synthesis

We provided a narrative synthesis of the summary effect estimates outlining the incidence and CVD-related mortality risk after a cancer diagnosis, together with 95% confidence intervals (CIs), measures of heterogeneity, and p-values where available and relevant. Forest plots were used to visualise the relative measures.

The extent of overlap among reviews included in our umbrella review was quantified using the corrected covered area [31].

$$\:CCA=\frac{N-r}{\left(rxc\right)-r}$$

Where N denotes the total number of publications (including duplicates), r the number of unique studies, and c the number of reviews. The scores were categorised as slight (< 5), moderate (6–10), high (11–15), and very high (> 15) overlap.

## Results

### Review selection

**Fig. 1:**
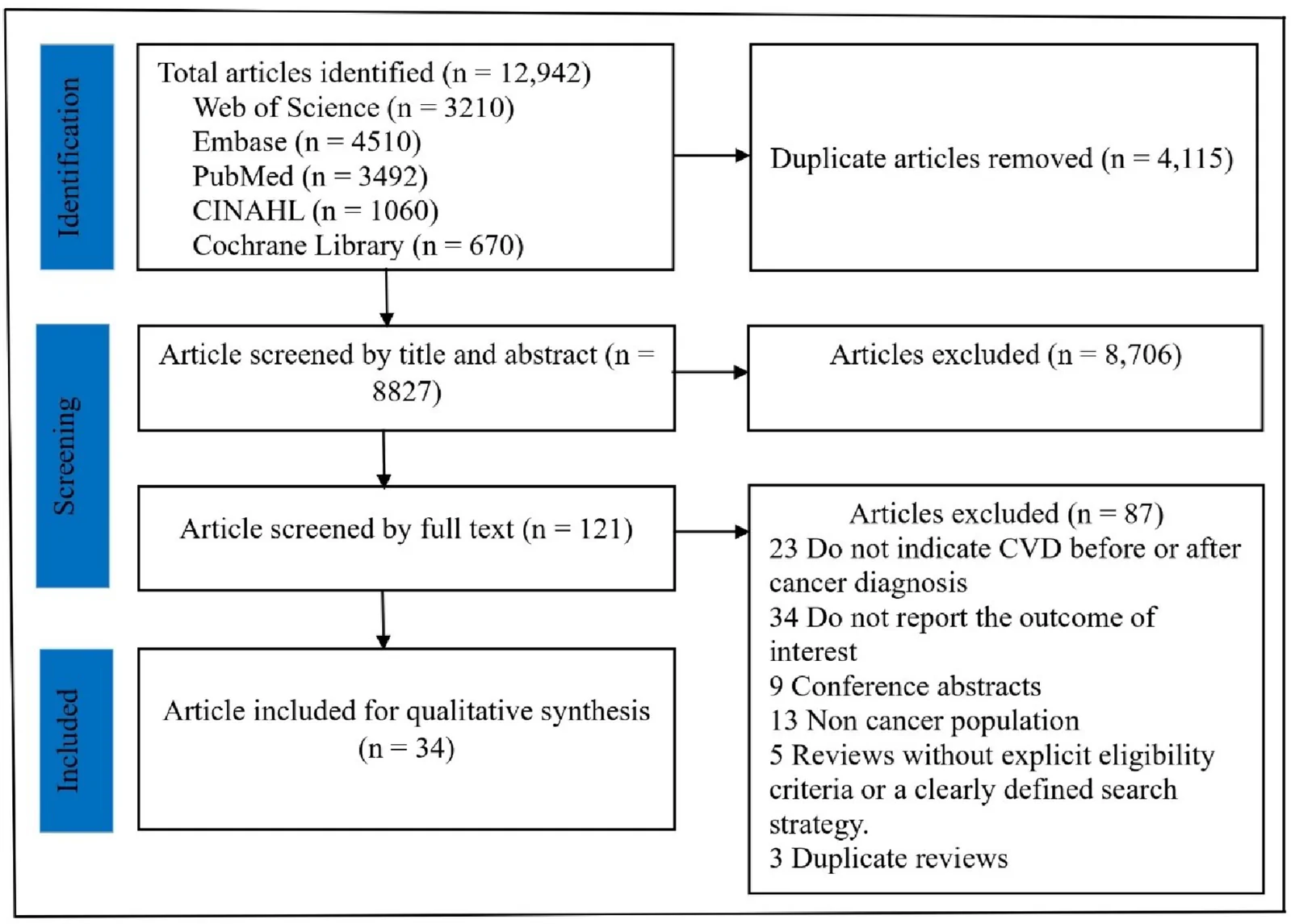
PRISMA flow diagram

A total of 12,942 reviews were identified, of which 4,115 were duplicates. After screening the titles and abstracts of the remaining 8,827 reviews, 121 full-text reviews were assessed for eligibility. Of these, 34 systematic reviews and/or meta-analyses met the inclusion criteria [15–17, 19–26, 32–54], (Fig. 1) including a total of 1,314 primary studies with only a slight overlap (0.23%) (Supplemental Tables 1 and 2).

### Characteristics

The characteristics of the reviews are presented in Table 1. We included three systematic reviews and 31 meta-analyses published between 2006 and 2025 and collectively included 1,218 non-overlapping primary studies (5–146 studies each). Most of these publications specified the types of primary studies included and used the Newcastle–Ottawa Scale (NOS) for quality appraisal.

**Table 1:** Characteristics of the included reviews

Author	Year	Type of review	Database searched	Review period	Number of included studies	Study design	Quality appraisal tool	AMSTAR 2
Shin Ng [23]	2014	MA	Medline, CINAHL, Scopus	January 01, 2000, to July 16, 2023	136	Cohort, case control, case series	NOS	Moderate
Lun [33]	2022	MA	Medline, Embase	January 1980 to June 2021	41	Cohort	NOS	Moderate
Horsted [34]	2012	MA	Medline, Embase	January 1, 1966, to July 14, 2011	46	Cohort	NA	Critically low
Suleman [35]	2024	MA	Medline, Embase, and CINAHL	January 1990 to March 2022	46	Clinical trial, cohort	JBI	Low
Caruso [16]	2010	MA	PubMed, Medline, Embase, and Cochrane Library	1985 to 2010	18	Cohort	NA	Critically low
Malgor [32]	2012	SR	Medline, Web of Science, Cochrane Library	NA	26	RCT, cohort, case control	NA	Critically low
Yuan [36]	2019	MA	PubMed, Web of Science	To June 2017	5	Case control, cohort	NOS	Low
Catueno [15]	2022	SR	PubMed, Cochrane Library, Google Scholar	January 1, 1995, to February 1, 2022	8	Cohort	JBI	Critically low
Weeks [37]	2020	MA	PubMed, Embase, and CINAHL	To April 17, 2019	20	Cohort	NOS	Moderate
Galimzhanov [20]	2023	MA	PubMed, Scopus, Web of Science	To March 23, 2022	142	RCT, cohort	NOS	Moderate
Bao [38]	2023	MA	PubMed, Embase, Web of Science	To October 31, 2022.	16	Cohort, case control, cross-sectional	NOS	Low
Boyne [24]	2018	MA	Embase, Medline, and CINAHL	To November 22, 2016.	27	Cohort	NOS	Moderate
Caruso [19]	2006	MA	PubMed	1970 to 2006	17	RCT, observational comparative studies	NA	Critically low
Zhang [39]	2020	MA	PubMed, Embase, Cochrane Library	To February 8, 2020	20	Cohort	NOS	Moderate
Balomenakis [40]	2023	MA	PubMed, Medline, Web of Science, Scopus, Cochrane Library, OpenGrey, EThOS	To July 22, 2022	17	Cohort, RCT	CLARITY group tool	Moderate
Wu [41]	2017	MA	PubMed, Embase, Web of Science	1990 to 2014	144	NA	NOS	Low
Kostopoulos [42]	2021	MA	Medline, Cochrane Library, clinicaltrials.gov	NA	6	Case control, cohort	NOS	Moderate
Monaghan [43]	2024	MA	PubMed, Scopus, CINAHL	To April 2023	146	NA	ROBINS-E	Low
Qian [22]	2015	MA	PubMed, Embase, Web of Science, Google Scholar	To July 8, 2015	9	Observational	NA	Critically low
Pastori [26]	2023	MA	Medline, Cochrane Library	To September 7, 2021	15	Observational and RCT	ROBINS-I, RoB 2, NOS	Moderate
Khan [44]	2017	MA	Medline, Embase	To January week 2, 2015	8	Cohort and RCT	NA	Critically low
Wang [45]	2023	MA	PubMed, Web of Science, Cochrane Library	To 2021	70	Single-arm studies, cohort	NHLBI and NOS	Moderate
Mauro [46]	2022	MA	PubMed, Medline, Embase	January 2005 to August 2020	22	RCT, observational	ROBINS-I tool and ROB 2 tool	Low
Stone [47]	2019	MA	Embase, Medline, and CINAHL	To June 7, 2018	22	Cross-sectional, cohort	NOS	Moderate
Pergialiotis [48]	2024	MA	Medline, Scopus, Clinicaltrials.gov, Cochrane Library, Google Scholar	To August 15, 2023	14	NR	NOS	Moderate
Dongchen [17]	2023	MA	Embase, PubMed, and Cochrane Library	To May 2022	10	Case control, cohort	NOS	Moderate
Govsyeyev [49]	2021	MA	PubMed, Embase, Cochrane Library	NA	7	Cohort	NOS	Critically low
Yang [21]	2024	MA	PubMed, Web of Science, Embase, Cochrane Library	To November 30, 2023	54	NA	NOS	Moderate
Wang [50]	2025	MA	PubMed, Embase, Scopus	To March 10, 2024	22	Cohort	NOS	Moderate
Hua [51]	2019	MA	PubMed, Embase, Cochrane Library, China National Knowledge Infrastructure, Wanfang	To February 2019	16	Case control, cohort	NOS	Low
Veiga [54]	2023	MA	PubMed, Virtual Health Library, Cochrane Library	2013 to 2023	36	Case control, cohort	JBI	Moderate
Roule [53]	2020	MA	Medline, Cochrane Library	To 2018	9	NA	NOS	Low
Betts [52]	2024	NMA	Embase, Medline, Cochrane Library	January 1, 2005, to February 15, 2022	30	Cohort	NA	Critically low
Harry [25]	2025	SR	Medline, Embase, Cochrane Library	After January 1, 2000	124	NA	Cochrane risk-of-bias assessment tool	Low

NOS: Newcastle-Ottawa Scale, NA: Not applicable, NMA: Network meta-analysis, MA: Meta-analysis, ROBINS-E: Risk of Bias In Non-randomized Studies of Exposure, NHLBI: National Heart, Lung, and Blood Institute

Analysis of the methodological quality using AMSTAR-2 revealed that fifteen reviews [17, 20, 21, 23, 26, 32–41] were assessed as having moderate methodological quality, ten reviews were rated as low quality [25, 32, 42–49], and nine reviews were of critically low quality [15, 16, 19, 22, 50–54] (Table 1; Supplemental Table 3).

### Incidence of CVD after a cancer diagnosis

The incidence of CVD following a cancer diagnosis was reported in three systematic reviews [15, 25, 50] and 26 meta-analyses [16, 17, 19–22, 26, 33–43, 45–50, 52, 54]. Across the reviews, the reported incidence of CVD following a cancer diagnosis ranged from 3.6%[32] to 9.7%[25]. The pooled incidence estimates reported in the meta-analyses ranged from 3% [21] to 13.7% [54]. Eight meta-analyses compared the incidence of CVD between cancer patients and individuals without cancer [20, 22, 26, 34, 36, 38, 43, 51]. These analyses consistently showed that cancer patients had a higher risk of CVD, with relative risks (RR) ranging from 11% to 132% higher than those observed in cancer-free populations.

**Fig. 2:**
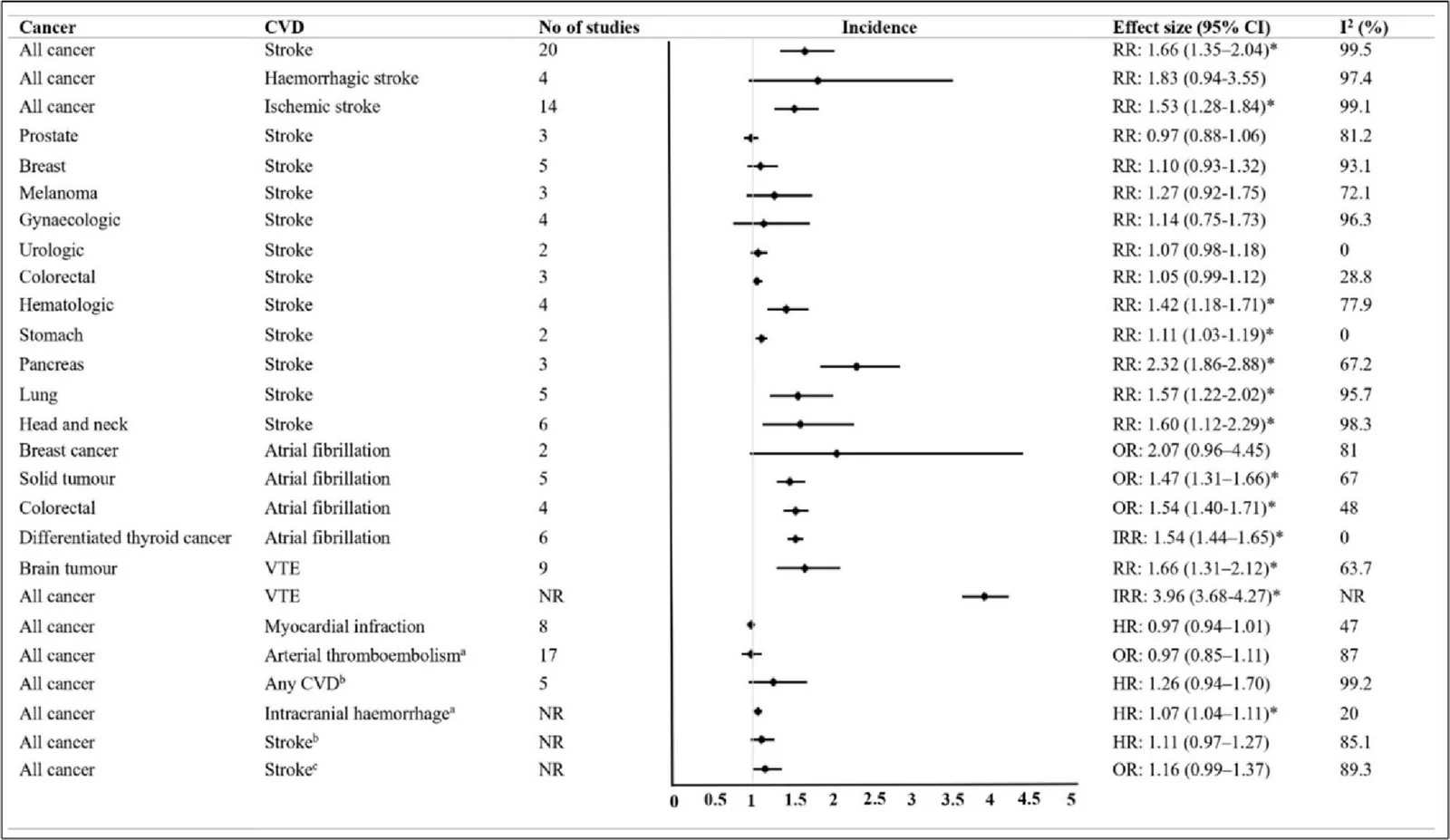
Forest plot showing the results of meta-analyses reporting the risk of CVD after a cancer diagnosis compared with cancer-free individuals New CVD in cancer patients with preexisting ^a^ atrial fibrillation, ^b^ myocardial infarction, ^c^ acute myocardial infarction. IRR: incidence rate ratio, NR: not reported, OR: odds ratio, RR: relative risk, HR: hazard ratio. *p-value: significant

Several reviews also reported the relative risks and/or incidence rates of specific cardiovascular events following a cancer diagnosis, including stroke, atrial fibrillation (AF), thrombosis, venous thromboembolism (VTE), arterial thromboembolism, pulmonary embolism (PE), MI, and other cardiovascular conditions. Some reviews further reported that individuals with pre-existing CVD developed additional forms of CVD after a cancer diagnosis (Fig. 2; Supplemental Tables 4 and 5).

### Stroke

Nine systematic reviews reported stroke incidence among cancer patients [15, 17, 20, 26, 32, 34, 37, 40, 46]. Among paediatric cancer patients, stroke incidence ranged from 0.47% to 2.9% across eight studies [15]. In adults, the pooled incidence of stroke within the first year after cancer diagnosis was 1.4% (95% CI: 0.9–2.2%; I² = 99.92%; *p* < 0.001) [33]. Stroke incidence also varied by subtype, with 1.3% for ischemic stroke (95% CI: 1.0–1.8%) and 0.3% for intracerebral haemorrhage (ICH) in the first year after diagnosis [32]. A meta-analysis of 20 studies showed that cancer survivors had a 66% higher risk of stroke compared with individuals without cancer [34] with a subgroup analysis of 14 studies showing a 53% higher risk specifically for ischemic stroke (RR = 1.53, 95% CI: 1.28–1.84; *p* < 0.001) [39]. Stroke risk also varied by cancer type, with significantly higher risks reported for head and neck, lung, pancreatic, stomach cancers, and haematological malignancies [34].

### Atrial fibrillation

Four systematic reviews reported the incidence of AF after cancer diagnosis [36, 43, 44, 47]. A meta-analysis of 12 studies estimated an annualised AF incidence of 0.7% within the first year after cancer treatment (95% CI: 0.1–0.98%)[38]. Among patients with differentiated thyroid cancer, AF incidence was 4.86 per 1,000 person-years, with a 54% higher risk compared with individuals without cancer (Incidence Rate Ratio (IRR) = 1.54; 95% CI: 1.44–1.65) [36].

In breast cancer patients, the 12-month incidence of AF was 0.9% following treatment [44], with pooled estimates suggesting an overall AF incidence of about 3% across studies [47].

Cancer patients with solid tumours also had a 47% higher risk of AF compared with non-cancer populations (Odds Ratio (OR) = 1.47; 95% CI: 1.31–1.66), with the highest risk observed within the first 90 days after cancer diagnosis (OR = 7.62; 95% CI: 3.08–18.88) [43].

### Thrombosis

Two reviews reported the incidence of thrombosis among cancer patients [16, 19]. Among lymphoma patients, thrombosis incidence was 6.5% in non-Hodgkin lymphoma and 4.7% in Hodgkin lymphoma, while central nervous system (CNS) lymphoma showed a markedly higher incidence of 48.1% [16]. In lymphoblastic leukemia, the overall thrombosis incidence was 5.2%, including 2.9% CNS thrombosis and 2.3% non-CNS thrombosis [19].

### VTE

Eleven reviews reported VTE incidence following cancer diagnosis [21, 22, 33, 37, 39, 41, 42, 45, 46, 51, 54]. Overall, 3.1% of cancer patients developed VTE within the first year [54], with a pooled incidence of 43 per 1,000 person-years, corresponding to an almost four-fold increased risk compared with the general population (IRR = 3.96) [34].

VTE incidence varied by cancer type, ranging from 12 per 1,000 person-years in prostate cancer to 116 per 1,000 in brain cancer [51], and was 6% in lung cancer [21], 5.5% in essential thrombocythemia [37], 11% in primary CNS lymphoma [42], and 13.7% in malignant neoplasms [41]. Advanced-stage ovarian cancer patients had a 2.7-fold higher risk of VTE than early-stage patients [33].

Brain tumours carried particularly high VTE risk, including glioma (RR = 1.68), high-grade glioma (RR = 1.70), and glioblastoma multiforme (RR = 1.74)[22]. VTE incidence in leukemia ranged from 3% in chronic lymphoblastic leukemia to 13% in chronic myeloid leukemia [45].

By subtype, essential thrombocythemia patients experienced peripheral (2.9%), deep (1.6%), abdominal (0.8%), and cerebral vein thromboses (0.2%) [45], while internal jugular vein thrombosis occurred in 12% of head and neck cancer patients [46].

### Arterial thromboembolism

Two reviews reported the incidence of arterial thromboembolism in cancer patients [37, 46]. Among patients with head and neck cancer, the incidence of arterial thrombosis was 1.9% (95% CI: 0.1–9.0%) [43]. A meta-analysis of 60 studies reported an arterial thrombosis incidence of 13.4% (95% CI: 12.0–14.8%; I² = 93%) among patients with essential thrombocythemia [37].

### Pulmonary embolism

Four reviews reported PE incidence in cancer patients [21, 37, 46, 50]. Among lung cancer patients, PE incidence ranged from 3% to 3.6% [32], while a meta-analysis of 34 studies estimated it at 3% (95% CI: 2%–4%; I² = 100%, *p* = 0.01) [21]. In head and neck cancer, PE occurred in 0.6%, while in essential thrombocythemia, the incidence was 0.3% [45].

### Other CVDs

In a meta-analysis of eight studies involving patients with cancer, the pooled HR for MI incidence compared to the cancer-free individuals was 0.97 (95% CI: 0.94–1.01; I² = 47%) [26]. A pooled analysis of 19 studies among breast cancer patients revealed that the incidence of MI was 1.98 per 1,000 person-years (95% CI: 1.24–3.16) [20]. The incidence of MI among patients with head and neck cancer was 2.0% (95% CI: 1.6–2.5%) [43], while a meta-analysis of 25 studies reported an incidence of 2.4% (95% CI: 1.9–3.0%; I² = 59%) in patients with essential thrombocythemia [37].

Lymphoma survivors have an increased incidence of CVD [38]. Patients with Hodgkin lymphoma had significantly increased risks for several cardiovascular conditions after 5 years of diagnosis, including cardiac dysrhythmia (Standardised incidence ratio (SIR = 3.23, 95% CI: 2.23–4.68; I² = 93.0%, *n* = 4 studies), coronary heart disease (SIR = 2.54, 95% CI: 1.95–3.31; I² = 97.2%, *n* = 11 studies), myocardial disease (SIR = 3.95, 95% CI: 2.48–6.27; I² = 97.1%, *n* = 7 studies), pericardial disease (SIR = 10.67, 95% CI: 7.75–14.69; I² = 63.3%, *n* = 3 studies), and valvular heart disease (SIR = 13.10, 95% CI: 7.41–23.16; I² = 96.2%, *n* = 5 studies). Similarly, Non-Hodgkin Lymphoma survivors have an increased incidence of most cardiovascular diseases, except coronary heart disease (SIR = 1.14, 95% CI 0.95–1.37, *n* = 4 studies) [38]. The incidence of heart failure and coronary artery disease among breast cancer patients was 4.44 (95% CI: 3.33–5.92; *n* = 51 studies) and 4.29 (95% CI: 3.09–5.94; *n* = 27 studies) per 1,000 person-years, respectively [20].

### Incidence of new CVD among cancer patients with preexisting CVD

Cancer patients with pre-existing CVD are at risk of developing additional cardiovascular events following a cancer diagnosis. Several reviews have reported the incidence of new thromboembolic and cerebrovascular events in this population [17, 26, 32, 40]. A meta-analysis of five studies found that cancer patients with MI had an increased risk of major adverse cardiovascular and cerebrovascular events compared with those without cancer, although this association was not statistically significant (HR = 1.26; 95% CI: 0.94–1.70; I² = 99.2%; *p* = 0.126) [17].

The incidence of stroke among cancer patients with pre-existing CVD varies by cancer type. Among patients with atrial fibrillation (AF), stroke incidence ranged from 0.6% (95% CI, 0.5–0.6%) in those with colorectal cancer to 5.3% (95% CI, 0.8–27.5%; I² = 99.83%; *p* = 0.00) in those with head and neck cancer. The one-year incidence of ischaemic stroke among AF patients was 3.3% (95% CI, 2.4–4.6%; I² = 29.7%) [33]. Among AF patients, those with cancer had a 7% higher risk of intracranial haemorrhage compared with cancer-free AF patients (HR, 1.07; 95% CI, 1.04–1.11; I² = 20%) [26]. Although not statistically significant, cancer patients with MI had a slightly higher risk of stroke compared with patients without cancer (HR, 1.11; 95% CI, 0.97–1.27; I² = 85.1%) [17]. Similarly, cancer patients with acute MI were at a higher risk of stroke than those without cancer (OR, 1.16; 95% CI, 0.99–1.37; I² = 89.3%) [50].

### CVD-related mortality after a cancer diagnosis

#### Cardiovascular disease mortality

**Fig. 3:**
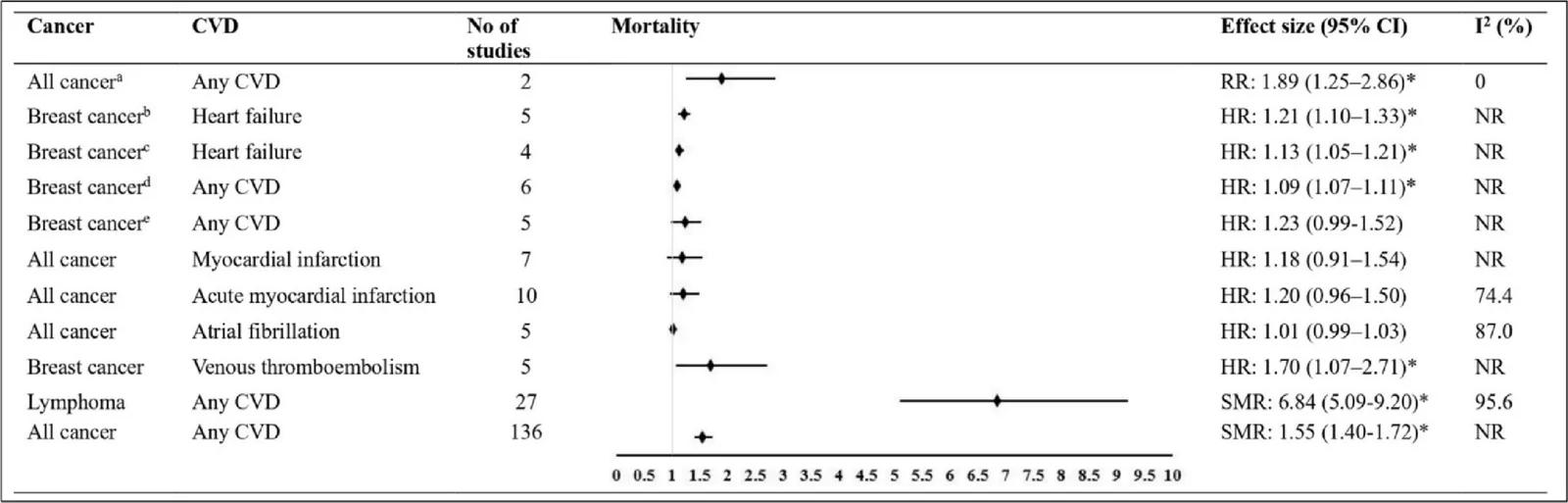
Forest plot showing the results of meta-analyses reporting CVD-related mortality after a cancer diagnosis compared with cancer-free individuals. Mortality ^a^ within one year, ^b^ two years, ^c^ three years, ^d^ five years, ^e^ eight to eleven years after cancer diagnosis. NR: not reported, RR: relative risk, HR: hazard ratio, SMR: standardised mortality ratio. *p-value: significant

Mortality risk from CVD after a cancer diagnosis was reported in a single review [18] and nine meta-analyses [17, 20, 23, 24, 26, 40, 42, 49, 52]. Eight meta-analyses reported CVD-related mortality after cancer diagnosis compared with individuals without cancer [17, 20, 23, 24, 26, 40, 49, 52]. These analyses consistently showed that cancer patients had a significantly higher risk of CVD-related death, with hazard ratios ranging from 9% to 70% higher than those observed in cancer-free populations (Fig. 3, Supplemental Tables 6 and 7).

A meta-analysis of two studies revealed that cancer patients had a higher risk of one-year cardiovascular death compared to cancer-free individuals (RR = 1.89; 95% CI: 1.25–2.86; I² = 0%) [53]. Similarly, a large meta-analysis of 136 studies reported a 55% increased risk of CVD-related death among cancer patients compared with the general population (Standardised Mortality Ratio (SMR) = 1.55, 95% CI: 1.40–1.72, *p* < 0.001) [23].

#### Mortality from CVD subtypes

Three reviews examined the risk of CVD mortality in cancer patients with specific cardiovascular conditions [17, 26, 40]. A meta-analysis of MI patients reported that cancer patients had a higher risk of CVD mortality during follow-up compared with those without cancer (HR = 1.31, 95% CI: 0.97–1.78) [17]. Another meta-analysis found that cancer patients with acute MI had a higher risk of in-hospital mortality due to cardiovascular complications (OR = 2.06, 95% CI: 1.17–3.65) [17]. In contrast, a pooled analysis of ten studies reported no significant increase in cardiovascular-related mortality (HR = 1.20, 95% CI: 0.96–1.50), and a meta-analysis of AF patients found similar cardiovascular mortality between cancer and non-cancer patients (HR = 1.01, 95% CI: 0.99–1.03) [26].

CVD-related mortality varied by cancer type. A meta-analysis of 136 studies reported SMRs from 1.14 (testicular cancer) to 2.82 (brain/CNS tumours). Breast cancer patients had a higher risk within five years post-diagnosis (HR = 1.09). Lymphoma survivors showed markedly elevated risk: Hodgkin lymphoma (SMR = 7.31) and non-Hodgkin lymphoma (SMR = 5.35) [24].

Cancer patients also show increased mortality from specific CVD subtypes. Risk of heart disease mortality (SMR) ranged from 1.13 (gynaecological cancers) to 2.46 (haematological malignancies), while cerebrovascular disease mortality was elevated (1.56) [23]. Among breast cancer patients, risk of death (HR) from heart failure within 2 years was 1.21 and from AF within 3 years was 1.13 [20]. Stroke mortality (SMR) varied from 1.25 (urological cancers) to 5.95 (brain/CNS tumours) [23], and PE-related mortality in primary CNS lymphoma was 4.05% [35].

## Discussion

To the best of our knowledge, this is the first comprehensive synthesis of evidence from systematic reviews and meta-analyses that addresses the incidence and mortality associated with CVD in patients with cancer. The findings showed that cancer patients faced significantly higher risks of CVD (e.g., RRs ranging from 1.11 to 2.32) and CVD-related mortality (e.g., HRs ranging from 1.09 to 1.70). Considerable variation in the incidence of CVD and CVD-related mortality was evident across cancer and CVD subtypes. Notably, patients with pre-existing CVD developed new-onset CVD after a cancer diagnosis, reflecting both disease progression and heightened cardiovascular vulnerability. Although the findings consistently suggest an increased cardiovascular burden among cancer patients, they should be interpreted with caution because none of the included reviews was rated as high methodological quality. This limits confidence in the available evidence and highlights the need for more rigorous systematic reviews and high-quality primary studies.

Several mechanisms may contribute to this elevated risk, including shared factors, such as smoking, obesity, and aging, systemic inflammation, endothelial dysfunction and the direct cardiotoxic effects of cancer treatments [6, 7, 9, 10, 12]. Additionally, cancer-associated hypercoagulability and oxidative stress may further accelerate vascular damage [6, 10, 12]. The physiological burden of cancer, compounded by the emotional and physical strain of treatment, imposes further stress on the cardiovascular system [55]. Pre-existing CVD may limit the use of aggressive cancer therapies, leading to suboptimal oncologic outcomes and additional cardiovascular complications [56]. Furthermore, coincident cancer and CVD may lead to fragmented care, and early signs of CVD may be missed if critical care is not well coordinated [57].

The incidence of CVD among cancer patients varies based on cancer type and the specific CVD subtype. For example, hematologic malignancies, such as lymphoma and leukemia, were strongly associated with cardiomyopathy and HF and often linked to anthracycline-based chemotherapy [58], while breast cancer patients were at an elevated risk of CVD from both anthracycline [59] and HER2-targeted treatments [60]. Thoracic radiotherapy for lung or esophageal cancers has been linked to a higher risk of pericardial disease and valvular dysfunction [61]. Among head and neck cancer survivors, radiation-induced vascular injury has been associated with an elevated risk of stroke [62]. These variations suggest that both tumour biology [10, 12] and specific cancer treatment modalities can significantly influence cardiovascular risk, highlighting the need for personalised monitoring and prevention strategies [63].

Preventive strategies are critical to mitigating the risk of developing CVD in this patient cohort. Evidence supports the need for baseline cardiovascular assessment [64], and, where indicated, the use of cardioprotective medications [65] and critical lifestyle modifications [66]. For patients with pre-existing CVD, proactive management is essential to prevent disease progression or complications, particularly in those receiving cardiotoxic cancer therapies [67].

Cancer survivors face an increased risk of CVD-related mortality compared to the general population, with the level of risk varying by cancer type and specific cardiovascular conditions. Cancer patients with comorbidities tend to have poorer survival outcomes than their comparatively healthier counterparts [68]. This increased mortality risk may be multifactorial, resulting from cumulative exposures to biological factors and cancer therapies [69]. Demographic and clinical factors also influence CVD-related mortality in this population [70]. Delays in cardiovascular care or limited access due to the prioritisation of cancer treatment may also contribute to poorer outcomes [40]. Additionally, cancer survivors may struggle to maintain healthy habits in daily life [71]. Unfortunately, adherence to CVD-related medications is generally low, with compliance declining over time [72]. An interdisciplinary approach focused on early prevention, risk reduction and critical cardio-oncology principles may reduce CVD-related mortality [73].

The comprehensive evidence generated demonstrates an increased risk of CVD and related adverse outcomes among cancer patients, highlighting the importance of carefully evaluating cardiovascular health in this population. These findings encourage cardio-oncologists to consider individual-level risk factors and support the development of robust cardiovascular risk prediction. Future research should prioritise well-designed studies to better delineate the risk of CVD in this patient group.

This umbrella review has some limitations. The quality of the primary studies included in each systematic review and meta-analysis relied on the assessments reported in the respective reviews rather than independent evaluation. As inherent to umbrella reviews, we relied on the quality assessments reported in the included systematic reviews and did not independently appraise the methodological quality or risk of bias of the individual primary studies. The certainty of the evidence was not assessed, although the methodological quality of the included reviews was evaluated using AMSTAR 2. The systematic reviews and meta-analyses included in this umbrella review primarily focused on the incidence of CVD and/or CVD-related mortality and did not include reviews examining cardiovascular adverse effects associated with specific cancer treatments. Therefore, when interpreting the findings for clinical practice, it is important to recognise that CVD related to specific cancer treatments was not examined in this review. Considerable heterogeneity across the included reviews in cancer types, CVD definitions, study populations, follow-up periods, outcome ascertainment, and confounder adjustment limited direct comparison and precluded a pooled analysis. Nevertheless, the conclusions drawn from the available evidence have important implications for clinical practice.

## Conclusions

In this umbrella review, the findings support the existing understanding that cancer patients have a higher risk of CVD and CVD-related mortality than cancer-free population; however, these findings should be interpreted with caution, given the generally low or moderate methodological quality of the included reviews. Nevertheless, the findings highlight the importance of integrated cardio-oncology care, improved risk prediction, and targeted prevention and treatment strategies to improve long-term outcomes.

## Supplementary Information

Below is the link to the electronic supplementary material.

Supplementary Material 1

Supplementary Material 2

Supplementary Material 3

## Data Availability

No datasets were generated or analysed during the current study.

## Abbreviations

AF: atrial fibrillation
CI: confidence intervals
CVD: cardiovascular disease
HR: hazard ratio
IRR: incidence rate ratio
MI: myocardial infarction
OR: odds ratio
PE: pulmonary embolism
RR: relative risk
SIR: standardised incidence ratio
SMR: standardised mortality ratio
VTE: venous thromboembolism
