# Supplementary material for: Incidence of and mortality risk from cardiovascular diseases after a cancer diagnosis: An umbrella review
Source: Clin Exp Med. 2026 Aug 17;26(1):375. doi: 10.1007/s10238-026-02295-0 (PMC13759700; doi:10.1007/s10238-026-02295-0)
Supplement: Supplementary file 3 [file 10238_2026_2295_MOESM3_ESM.docx]

**Supplementary Content**

**Sewunet Admasu Belachew, PhD; Solomon Ahmed Mohammed, Msc; Gail Garvey, PhD.** **Incidence of and mortality risk from cardiovascular diseases after a Cancer Diagnosis: An Umbrella Review**

**Supplementary Table 1.** Search terms and strategy

**Supplementary Table 2.** Excluded overlapping reviews

**Supplementary Table 3.** Quality appraisal results of included reviews using the AMSTAR-2 Tool

**Supplementary Table 4.** Incidence of CVD after a cancer diagnosis

**Supplementary Table 5**. CVD incidence follow-up period and outcomes

**Supplementary Table 6.** CVD-related mortality after a cancer diagnosis

**Supplementary Table 7**. CVD mortality follow-up period and outcome

**Supplementary Table 1.** Search terms and strategy

**Databases**

1) PubMed
2) Embase (Elsevier)
3) CINHAL (EBSCOhost)
4) Cochrane Library
5) Web of Science

| **PubMed** | | |
| --- | --- | --- |
| **Sr no** | **Search** | **Results** |
| 1 | "incidence"[Title/Abstract] OR "incidence"[MeSH Terms] OR "morbidity"[Title/Abstract] OR "morbidity"[MeSH Terms] OR "mortality"[Title/Abstract] OR "mortality"[MeSH Terms] OR survival [Title/Abstract] OR survival [MeSH] OR "death*"[Title/Abstract] OR "death"[MeSH Terms] OR "fatal*"[Title/Abstract] OR prognosis [MeSH] | 5,778,156 |
| 2 | "cardiovascular disease"[Title/Abstract] OR "cardiovascular diseases"[MeSH Terms] OR "CVD"[Title/Abstract] OR "cardiovascular outcome*"[Title/Abstract] OR "cardiovascular event*"[Title/Abstract] OR "cardiovascular complication*"[Title/Abstract] OR "cardiac disease"[Title/Abstract] OR "cardiac outcome*"[Title/Abstract] OR "cardiac event*"[Title/Abstract] OR "cardiac complication*"[Title/Abstract] OR "cardiotoxicit*"[Title/Abstract] OR "cardiotoxic"[Title/Abstract] OR "cardiomyopath*"[Title/Abstract] OR "systolic dysfunction"[Title/Abstract] OR "myocardial dysfunction"[Title/Abstract] OR "cardiac dysfunction"[Title/Abstract] OR "CTRCD"[Title/Abstract] OR "arrhythmia*"[Title/Abstract] OR "QT prolongation"[Title/Abstract] OR "myocarditis"[Title/Abstract] OR "hypertension"[Title/Abstract] OR "vascular toxicit*"[Title/Abstract] OR "coronary"[Title/Abstract] OR "ischemic"[Title/Abstract] OR "ischaemic"[Title/Abstract] OR "myocardial infarction"[Title/Abstract] OR "stroke"[Title/Abstract] OR "cerebrovascular"[Title/Abstract] OR "thromboembo*"[Title/Abstract] OR "thrombosis"[Title/Abstract] OR "VTE"[Title/Abstract] OR "cardiotoxicity"[MeSH Terms] OR "peripheral arterial disease"[MeSH Terms] OR "heart valve diseases"[MeSH Terms] OR "heart failure"[MeSH Terms] OR "coronary artery disease"[MeSH Terms] OR "myocardial ischemia"[MeSH Terms] OR "coronary artery disease"[MeSH Terms] OR "stroke"[MeSH Terms] OR "venous thromboembolism"[MeSH Terms] | 3,547, 514 |
| 3 | "cancer*"[Title/Abstract] OR "carcinoma*"[Title/Abstract] OR "tumor*"[Title/Abstract] OR "tumour*"[Title/Abstract] OR "malignan*"[Title/Abstract] OR "neoplasm*"[Title/Abstract] OR "oncology"[Title/Abstract] OR "Neoplasms"[MeSH Terms] | 5,461,305 |
| 4 | "systematic review"[Title/Abstract] OR "systematic literature review"[Title/Abstract] OR "integrative-review"[Title/Abstract] OR "integrative research review"[Title/Abstract] OR "integrative review"[Title/Abstract] OR "meta-synthesis"[Title/Abstract] OR "meta synthesis"[Title/Abstract] OR "meta-analysis"[Title/Abstract] OR "metaanalysis"[Title/Abstract] OR "meta analyses"[Title/Abstract] OR "metanalyses"[Title/Abstract | 511,354 |
| 5 | #1 AND #2 AND #3 AND #4 | 4,662 |
| 6 | Limit 5 to (meta-analysis, review, systematic review) | 4,082 |
| 7 | Limit 6 to humans | 3,574 |
| 8 | Limit 7 to English | 3,492 |
| **Embase (Elsevier)** | | |
| 1 | incidence:ti,ab OR 'incidence'/exp OR morbidity:ti,ab OR 'morbidity'/exp OR mortality:ti,ab OR 'mortality'/exp OR survival:ti,ab OR 'survival'/exp OR death*:ti,ab OR 'death'/exp OR fatal*:ti,ab OR 'prognosis'/exp | 7,079,431 |
| 2 | 'cardiovascular disease':ti,ab OR 'cardiovascular disease'/exp OR cvd:ti,ab OR 'cardiovascular outcome*':ti,ab OR 'cardiovascular event*':ti,ab OR 'cardiovascular complication*':ti,ab OR 'cardiac disease':ti,ab OR 'cardiac outcome*':ti,ab OR 'cardiac event*':ti,ab OR 'cardiac complication*':ti,ab OR cardiotoxicit*:ti,ab OR cardiotoxic:ti,ab OR cardiomyopath*:ti,ab OR 'systolic dysfunction':ti,ab OR 'myocardial dysfunction':ti,ab OR 'cardiac dysfunction':ti,ab OR ctrcd:ti,ab OR arrhythmia*:ti,ab OR 'qt prolongation':ti,ab OR myocarditis:ti,ab OR hypertension:ti,ab OR 'vascular toxicit*':ti,ab OR coronary:ti,ab OR ischemic:ti,ab OR ischaemic:ti,ab OR 'myocardial infarction':ti,ab OR stroke:ti,ab OR cerebrovascular:ti,ab OR thromboembo*:ti,ab OR thrombosis:ti,ab OR vte:ti,ab OR 'cardiotoxicity'/exp OR 'peripheral artery disease'/exp OR 'valvular heart disease'/exp OR 'heart failure'/exp OR 'coronary artery disease'/exp OR 'ischemic heart disease'/exp OR 'cerebrovascular accident'/exp OR 'venous thromboembolism'/exp | 6,261,604 |
| 3 | cancer*:ti,ab OR carcinoma*:ti,ab OR tumor*:ti,ab OR tumour*:ti,ab OR malignan*:ti,ab OR neoplasm*:ti,ab OR oncology:ti,ab OR 'neoplasm'/exp | 7,705,992 |
| 4 | 'systematic review':ti,ab OR 'systematic literature review':ti,ab OR 'integrative research review':ti,ab OR 'integrative-review':ti,ab OR 'integrative review':ti,ab OR 'meta-synthesis':ti,ab OR 'meta synthesis':ti,ab OR 'meta-analysis':ti,ab OR 'meta analysis':ti,ab OR 'metaanalysis':ti,ab OR 'meta analyses':ti,ab OR 'metanalyses':ti,ab | 620,979 |
| 5 | #1 AND #2 AND #3 AND #4 | 11,090 |
| 6 | #5 AND ('human'/de OR 'meta analysis'/de OR 'meta analysis topic'/de OR 'network meta analysis'/de OR 'systematic review'/de OR 'systematic review topic'/de) | 11,032 |
| 7 | #6 AND 'review'/it | 4,596 |
| 8 | #7 AND [english]/lim | 4,510 |
| **CINHAL (EBSCOhost)** | | |
| 1 | ((TI incidence OR AB incidence) OR (MH incidence+) OR (TI morbidity OR AB morbidity) OR (MH morbidity+) OR (TI mortality OR AB mortality) OR (MH mortality+) OR (TI survival OR AB survival) OR (MH survival+) OR (TI death* OR AB death*) OR (MH death+) OR (TI fatal* OR AB fatal*) OR (MH prognosis+)) | 1,382,538 |
| 2 | ((TI "cardiovascular disease" OR AB "cardiovascular disease") OR (MH "cardiovascular diseases+") OR (TI CVD OR AB CVD) OR (TI "cardiovascular outcome*" OR AB "cardiovascular outcome*") OR (TI "cardiovascular event*" OR AB "cardiovascular event*") OR (TI "cardiovascular complication*" OR AB "cardiovascular complication*") OR (TI "cardiac disease" OR AB "cardiac disease") OR (TI "cardiac outcome*" OR AB "cardiac outcome*") OR (TI "cardiac event*" OR AB "cardiac event*") OR (TI "cardiac complication*" OR AB "cardiac complication*") OR (TI cardiotoxicit* OR AB cardiotoxicit*) OR (TI cardiotoxic OR AB cardiotoxic) OR (TI cardiomyopath* OR AB cardiomyopath*) OR (TI "systolic dysfunction" OR AB "systolic dysfunction") OR (TI "myocardial dysfunction" OR AB "myocardial dysfunction") OR (TI "cardiac dysfunction" OR AB "cardiac dysfunction") OR (TI CTRCD OR AB CTRCD) OR (TI arrhythmia* OR AB arrhythmia*) OR (TI "QT prolongation" OR AB "QT prolongation") OR (TI myocarditis OR AB myocarditis) OR (TI hypertension OR AB hypertension) OR (TI "vascular toxicit*" OR AB "vascular toxicit*") OR (TI coronary OR AB coronary) OR (TI ischemic OR AB ischemic) OR (TI ischaemic OR AB ischaemic) OR (TI "myocardial infarction" OR AB "myocardial infarction") OR (TI stroke OR AB stroke) OR (TI cerebrovascular OR AB cerebrovascular) OR (TI thromboembo* OR AB thromboembo*) OR (TI thrombosis OR AB thrombosis) OR (TI VTE OR AB VTE) OR (MH cardiotoxicity+) OR (MH "heart valve diseases+") OR (MH "heart failure+") OR (MH "coronary arteriosclerosis+") OR (MH "myocardial ischemia+") OR (MH stroke+) OR (MH "venous thromboembolism+")) | 869,471 |
| 3 | (TI cancer* OR AB cancer*) OR (TI carcinoma* OR AB carcinoma*) (TI tumor* OR AB tumor*) OR (TI tumour* OR AB tumour*) OR (TI malignan* OR AB malignan*) OR (TI neoplasm* OR AB neoplasm*) OR (TI oncology OR AB oncology) OR (MH neoplasm+)) | 644, 435 |
| 4 | ((TI “systematic review” OR AB “systematic review”) OR (TI “systematic literature review” OR AB “systematic literature review”) OR (TI “integrative research review” OR AB “integrative research review”) OR (TI “integrative-review” OR AB “integrative-review”) OR (TI meta-synthesis OR AB meta-synthesis) OR (TI meta-analysis OR AB meta-analysis) OR (TI metaanalysis OR AB metaanalysis) OR (TI “meta analyses” OR AB “meta analyses”) OR (TI metanalyses OR AB metanalyses)) | 214,715 |
| 5 | #1 AND #2 AND #3 AND #4 | 1,071 |
| 6 | #7 limit to English | 1,060 |
| **Cochrane library** | | |
| 1 | (incidence):ti,ab,kw OR MeSH descriptor: [Incidence] explode all trees OR (morbidity):ti,ab,kw OR MeSH descriptor: [Morbidity] explode all trees OR (mortality):ti,ab,kw OR MeSH descriptor: [Mortality] explode all trees OR (survival):ti,ab,kw OR MeSH descriptor: [Survival] explode all trees OR (death*):ti,ab,kw OR MeSH descriptor: [Death] explode all trees OR (fatal*):ti,ab,kw OR MeSH descriptor: [Prognosis] explode all trees | 572,205 |
| 2 | (cardiovascular NEXT disease):ti,ab,kw OR MeSH descriptor: [Cardiovascular Diseases] explode all trees OR (CVD):ti,ab,kw OR (cardiovascular NEXT outcome*):ti,ab,kw OR (cardiovascular NEXT event*):ti,ab,kw OR (cardiovascular NEXT complication*):ti,ab,kw OR (cardiac NEXT disease):ti,ab,kw OR (cardiac NEXT outcome*):ti,ab,kw OR (cardiac NEXT event*):ti,ab,kw OR (cardiac NEXT complication*):ti,ab,kw OR (cardiotoxicit*):ti,ab,kw OR (cardiotoxic):ti,ab,kw OR (cardiomyopath*):ti,ab,kw OR (systolic NEXT dysfunction):ti,ab,kw OR (myocardial NEXT dysfunction):ti,ab,kw OR (cardiac NEXT dysfunction):ti,ab,kw OR (CTRCD):ti,ab,kw OR (arrhythmia*):ti,ab,kw OR (QT prolongation):ti,ab,kw OR (myocarditis):ti,ab,kw OR (hypertension):ti,ab,kw OR (vascular NEXT toxicit*):ti,ab,kw OR (coronary):ti,ab,kw OR (ischemic):ti,ab,kw OR (ischaemic):ti,ab,kw OR (myocardial NEXT infarction):ti,ab,kw OR (stroke):ti,ab,kw OR (cerebrovascular):ti,ab,kw OR (thromboembo*):ti,ab,kw OR (thrombosis):ti,ab,kw OR (VTE):ti,ab,kw OR MeSH descriptor: [Cardiotoxicity] explode all trees OR MeSH descriptor: [Peripheral Arterial Disease] explode all trees OR MeSH descriptor: [Heart Valve Diseases] explode all trees OR MeSH descriptor: [Heart Failure] explode all trees OR MeSH descriptor: [Myocardial Ischemia] explode all trees OR MeSH descriptor: [Stroke] explode all trees OR MeSH descriptor: [Venous Thromboembolism] explode all trees OR MeSH descriptor: [Coronary Artery Disease] explode all trees | 330,786 |
| 3 | (cancer):ti,ab,kw OR (carcinoma):ti,ab,kw OR (tumor):ti,ab,kw OR (tumour):ti,ab,kw OR (malignan*):ti,ab,kw OR (neoplasm*):ti,ab,kw OR (oncology):ti,ab,kw OR MeSH descriptor: [Neoplasms] explode all trees | 302,957 |
| 4 | (systematic NEXT review):ti,ab,kw OR ("systematic literature review"):ti,ab,kw OR (integrative-review):ti,ab,kw OR ("integrative research review"):ti,ab,kw OR (integrative NEXT review):ti,ab,kw OR (meta-synthesis):ti,ab,kw OR (meta NEXT synthesis):ti,ab,kw OR (metaanalysis):ti,ab,kw OR (metaanalysis):ti,ab,kw OR (meta NEXT analyses):ti,ab,kw OR (metaanalyses):ti,ab,kw | 28,180 |
| 5 | #1 AND #2 AND #3 AND #4 | 673 |
| **Web of Science** | | |
| 1 | (((((((TS=(incidence)) OR TS=(morbidity)) OR TS=(mortality)) OR TS=(survival)) OR TS=(death*))) OR TS=(fatal*)) | 5,142,804 |
| 2 | ((((((((((((((((((((((((((((((TS=(cardiovascular-disease)) OR TS=(CVD)) OR TS=(cardiovascular-outcome*)) OR TS=(cardiovascular-event*)) OR TS=(cardiovascular-complication*)) OR TS=(cardiac-disease)) OR TS=(cardiac-outcome*)) OR TS=(cardiac-event*)) OR TS=(cardiaccomplication*)) OR TS=(cardiotoxicit*)) OR TS=(cardiotoxic)) OR TS=(cardiomyopath*)) OR TS=(systolic-dysfunction)) OR TS=(myocardial-dysfunction)) OR TS=(cardiac-dysfunction)) OR TS=(CTRCD)) OR TS=(arrhythmia*)) OR TS=(QT-prolongation)) OR TS=(myocarditis)) OR TS=(hypertension)) OR TS=(vascular-toxicit*)) OR TS=(coronary)) OR TS=(ischemic)) OR TS=(ischaemic)) OR TS=(myocardial-infarction)) OR TS=(stroke)) OR TS=(cerebrovascular)) OR TS=(thromboembo*)) OR TS=(thrombosis)) OR TS=(VTE)) | 2,727,081 |
| 3 | (((((TS=(cancer*)) OR TS=(carcinoma*)) OR TS=(tumor*)) OR TS=(tumour*)) OR TS=(malignan*)) OR TS=(neoplasm*) | 5,472,930 |
| 4 | ((((((TS=(systematic review)) OR TS=(integrative review)) OR TS=(meta synthesis)) OR TS=(meta analysis)) OR TS=(meta analyses)) OR TS=(metaanalysis)) OR TS=(meta-analyses) | 992,903 |
| 5 | #1 AND #2 AND #3 AND #4 | 7,444 |
| 6 | #5 limit to review article | 3,263 |
| 7 | #6 limit to English | 3,210 |

**Supplementary Table 2.** Excluded overlapping reviews

| Sr. no | Study |
| --- | --- |
| 1 | Gernaat S, Ho P, Rijnberg N, Emaus M, Baak L, Hartman M, et al. Risk of death from cardiovascular disease following breast cancer: a systematic review. Breast cancer research and treatment 2017; 164:537-55. |
| 2 | Turner M, Murchie P, Derby S, Ong AY, Walji L, McLernon D, et al. Is stroke incidence increased in survivors of adult cancers? A systematic review and meta-analysis. Journal of Cancer Survivorship 2022;16(6):1414-48. |
| 3 | Kourlaba G, Relakis J, Mylonas C, Kapaki V, Kontodimas S, Holm MV, et al. The humanistic and economic burden of venous thromboembolism in cancer patients: a systematic review. Blood Coagulation & Fibrinolysis 2015;26(1):13-31. |

**Supplementary Table 3.** Quality appraisal results of included reviews using the AMSTAR-2 Tool

| Author | Q1 | Q2 | Q3 | Q4 | Q5 | Q6 | Q7 | Q8 | Q9 | Q10 | Q11 | Q12 | Q13 | Q14 | Q15 | Q16 | Overall |
| --- | --- | --- | --- | --- | --- | --- | --- | --- | --- | --- | --- | --- | --- | --- | --- | --- | --- |
| Shin Ng ^1^ | Yes | Yes | Yes | Yes | Yes | Yes | Partially yes | No | Yes | No | Yes | No | Yes | Yes | Yes | Yes | Moderate |
| Lun ^2^ | Yes | Yes | Yes | Yes | Yes | Yes | Partially yes | Partially yes | Yes | No | Yes | Yes | Yes | Yes | No | Yes | Low |
| Horsted ^3^ | Yes | No | Yes | Yes | Yes | Yes | Partially yes | Yes | No | No | Yes | No | No | Yes | No | Yes | Critically low |
| Suleman ^4^ | Yes | No | Yes | Yes | Yes | Yes | Partially yes | Yes | Yes | No | Yes | Yes | Yes | Yes | Yes | Yes | Low |
| Caruso ^5^ | Yes | No | Yes | Yes | Yes | No | Partially yes | Yes | No | No | Yes | No | No | Yes | No | Yes | Critically low |
| Malgor ^6^ | Yes | No | Yes | Partially yes | Yes | No | Partially yes | Partially yes | No | No | No MA | No MA | No | No | No MA | Yes | Critically low |
| Yuan ^7^ | Yes | No | Yes | Yes | Yes | No | Partially yes | Partially yes | Yes | No | Yes | Yes | Yes | Yes | Yes | Yes | Low |
| Catueno ^8^ | Yes | No | Yes | Partially yes | Yes | Yes | Partially yes | partially yes | Yes | No | No MA | No MA | No | No | No MA | No | Critically low |
| Weeks ^9^ | Yes | Yes | Yes | Yes | No | Yes | Partially yes | Partially yes | Yes | No | Yes | Yes | Yes | Yes | Yes | Yes | Moderate |
| Galimzhanov ^10^ | Yes | Yes | Yes | Yes | No | No | Yes | Partially yes | Yes | No | Yes | Yes | Yes | Yes | Yes | No | Moderate |
| Bao ^11^ | Yes | Yes | Yes | Yes | Yes | Yes | Partially yes | Partially yes | Yes | No | Yes | No | Yes | Yes | No | Yes | Low |
| Boyne ^12^ | Yes | Yes | Yes | Yes | Yes | No | Partially yes | Partially yes | Yes | No | Yes | Yes | Yes | Yes | Yes | Yes | Moderate |
| Caruso ^13^ | Yes | No | Yes | No | No | No | Yes | No | No | No | Yes | No | No | Yes | No | No | Critically low |
| Zhang ^14^ | Yes | Yes | Yes | Yes | Yes | Yes | Partially yes | Yes | Yes | No | Yes | Yes | Yes | Yes | Yes | Yes | Moderate |
| Balomenakis ^15^ | Yes | Yes | Yes | Yes | Yes | Yes | Partially yes | Partially yes | Yes | No | Yes | No | Yes | Yes | Yes | No | Moderate |
| Wu ^16^ | Yes | No | Yes | Yes | Yes | Yes | Partially yes | Yes | Yes | No | Yes | No | Yes | Yes | Yes | No | Low |
| Kostopoulos ^17^ | Yes | Yes | Yes | Yes | Yes | Yes | Partially yes | Yes | Yes | No | Yes | Yes | Yes | Yes | No | Yes | Moderate |
| Monaghan ^18^ | Yes | No | Yes | Yes | Yes | Yes | Partially yes | Partially yes | Yes | No | Yes | No | Yes | Yes | Yes | Yes | Low |
| Qian ^19^ | Yes | No | No | Yes | No | Yes | Partially yes | No | No | No | Yes | No | No | Yes | Yes | Yes | Critically low |
| Pastori ^20^ | Yes | Yes | Yes | Yes | Yes | No | Partially yes | Partially yes | Yes | No | Yes | No | Yes | Yes | Yes | Yes | Moderate |
| Khan ^21^ | Yes | No | Yes | Yes | Yes | Yes | Partially yes | Partially yes | No | No | Yes | No | No | No | No | Yes | Critically low |
| Wang ^22^ | Yes | Yes | Yes | Yes | No | No | Partially yes | Partially yes | Yes | No | Yes | No | Yes | No | Yes | Yes | Moderate |
| Mauro ^23^ | Yes | No | Yes | Yes | No | No | Partially yes | Partially yes | Yes | No | Yes | Yes | Yes | Yes | Yes | Yes | Low |
| Stone ^24^ | Yes | Yes | Yes | Yes | Yes | No | Partially yes | Partially yes | Yes | No | Yes | No | Yes | Yes | Yes | Yes | Moderate |
| Pergialiotis ^25^ | Yes | Yes | Yes | Yes | Yes | No | Partially yes | Partially yes | Yes | No | Yes | No | Yes | Yes | Yes | Yes | Moderate |
| Dongchen ^26^ | Yes | Yes | Yes | Yes | Yes | Yes | Partially yes | Partially yes | Yes | No | Yes | No | Yes | Yes | Yes | Yes | Moderate |
| Govsyeyev ^27^ | Yes | No | Yes | Yes | No | No | Partially yes | Partially yes | Yes | No | Yes | No | No | Yes | No | No | Critically low |
| Yang ^28^ | Yes | Yes | Yes | Yes | Yes | Yes | Partially yes | Partially yes | Yes | No | Yes | Yes | Yes | Yes | Yes | Yes | Moderate |
| Wang ^29^ | Yes | Yes | Yes | Yes | Yes | Yes | Yes | Partially yes | Yes | No | Yes | Yes | Yes | Yes | Yes | No | Moderate |
| Hua ^30^ | Yes | No | Yes | Yes | Yes | No | Partially yes | Partially yes | Yes | No | Yes | Yes | Yes | Yes | Yes | Yes | Low |
| Veiga ^31^ | Yes | Yes | Yes | Yes | Yes | Yes | Yes | Partially yes | Yes | No | Yes | Yes | Yes | Yes | Yes | No | Moderate |
| Roule ^32^ | Yes | No | Yes | Yes | Yes | No | Partially yes | Partially yes | Yes | No | Yes | Yes | Yes | Yes | Yes | Yes | Low |
| Betts ^33^ | Yes | Yes | Yes | Yes | Yes | Yes | Partially yes | Partially yes | No | No | Yes | Yes | No | Yes | Yes | Yes | Critically low |
| Harry ^34^ | Yes | No | Yes | Yes | Yes | No | Partially yes | Partially yes | Yes | No | No MA | No MA | Yes | Yes | No MA | Yes | Low |

MA: Meta-analysis

Q1. Did the research questions and inclusion criteria for the review include the components of PICO? For Yes: Population Intervention Comparator group Outcome Optional (recommended) Timeframe for follow-up Yes No

Q2. Did the report of the review contain an explicit statement that the review methods were established prior to the conduct of the review and did the report justify any significant deviations from the protocol? For Partial Yes: The authors state that they had a written protocol or guide that included ALL the following: review question(s) a search strategy inclusion/exclusion criteria a risk of bias assessment For Yes: As for partial yes, plus the protocol should be registered and should also have specified: a meta-analysis/synthesis plan, if appropriate, and a plan for investigating causes of heterogeneity justification for any deviations from the protocol Yes Partial Yes No

Q3. Did the review authors explain their selection of the study designs for inclusion in the review? For Yes, the review should satisfy ONE of the following: Explanation for including only RCTs OR Explanation for including only NRSI OR Explanation for including both RCTs and NRSI Yes No

Q4. Did the review authors use a comprehensive literature search strategy? For Partial Yes (all the following): searched at least 2 databases (relevant to research question) provided key word and/or search strategy justified publication restrictions (e.g. language) For Yes, should also have (all the following): searched the reference lists / bibliographies of included studies searched trial/study registries included/consulted content experts in the field where relevant, searched for grey literature conducted search within 24 months of completion of the review Yes Partial Yes No

Q5. Did the review authors perform study selection in duplicate? For Yes, either ONE of the following: at least two reviewers independently agreed on selection of eligible studies and achieved consensus on which studies to include OR two reviewers selected a sample of eligible studies and achieved good agreement (at least 80 percent), with the remainder selected by one reviewer. Yes No

Q6. Did the review authors perform data extraction in duplicate? For Yes, either ONE of the following: at least two reviewers achieved consensus on which data to extract from included studies OR two reviewers extracted data from a sample of eligible studies and achieved good agreement (at least 80 percent), with the remainder extracted by one reviewer. Yes No

Q7. Did the review authors provide a list of excluded studies and justify the exclusions? For Partial Yes: provided a list of all potentially relevant studies that were read in full-text form but excluded from the review For Yes, must also have: Justified the exclusion from the review of each potentially relevant study Yes Partial Yes No

Q8. Did the review authors describe the included studies in adequate detail? For Partial Yes (ALL the following): described populations described interventions described comparators described outcomes described research designs For Yes, should also have ALL the following: described population in detail described intervention in detail (including doses where relevant) described comparator in detail (including doses where relevant) described study’s setting timeframe for follow-up Yes Partial Yes No

Q9. Did the review authors use a satisfactory technique for assessing the risk of bias (RoB) in individual studies that were included in the review? RCTs For Partial Yes, must have assessed RoB from unconcealed allocation, and lack of blinding of patients and assessors when assessing outcomes (unnecessary for objective outcomes such as allcause mortality) For Yes, must also have assessed RoB from: allocation sequence that was not truly random, and selection of the reported result from among multiple measurements or analyses of a specified outcome Yes Partial Yes No Includes only NRSI NRSI For Partial Yes, must have assessed RoB: from confounding, and from selection bias For Yes, must also have assessed RoB: methods used to ascertain exposures and outcomes, and selection of the reported result from among multiple measurements or analyses of a specified outcome Yes Partial Yes No Includes only RCTs

Q10. Did the review authors report on the sources of funding for the studies included in the review? For Yes Must have reported on the sources of funding for individual studies included in the review. Note: Reporting that the reviewers looked for this information but it was not reported by study authors also qualifies Yes No

Q11. If meta-analysis was performed did the review authors use appropriate methods for statistical combination of results? RCTs For Yes: The authors justified combining the data in a meta-analysis AND they used an appropriate weighted technique to combine study results and adjusted for heterogeneity if present. AND investigated the causes of any heterogeneity Yes No No meta-analysis conducted For NRSI For Yes: The authors justified combining the data in a meta-analysis AND they used an appropriate weighted technique to combine study results, adjusting for heterogeneity if present AND they statistically combined effect estimates from NRSI that were adjusted for confounding, rather than combining raw data, or justified combining raw data when adjusted effect estimates were not available AND they reported separate summary estimates for RCTs and NRSI separately when both were included in the review Yes No No meta-analysis conducted

Q12. If meta-analysis was performed, did the review authors assess the potential impact of RoB in individual studies on the results of the meta-analysis or other evidence synthesis? For Yes: included only low risk of bias RCTs OR, if the pooled estimate was based on RCTs and/or NRSI at variable RoB, the authors performed analyses to investigate possible impact of RoB on summary estimates of effect. Yes No No meta-analysis conducted

Q13. Did the review authors account for RoB in individual studies when interpreting/ discussing the results of the review? For Yes: included only low risk of bias RCTs OR, if RCTs with moderate or high RoB, or NRSI were included the review provided a discussion of the likely impact of RoB on the results Yes No

Q14. Did the review authors provide a satisfactory explanation for, and discussion of, any heterogeneity observed in the results of the review? For Yes: There was no significant heterogeneity in the results OR if heterogeneity was present the authors performed an investigation of sources of any heterogeneity in the results and discussed the impact of this on the results of the review Yes No

Q15. If they performed quantitative synthesis did the review authors carry out an adequate investigation of publication bias (small study bias) and discuss its likely impact on the results of the review? For Yes: performed graphical or statistical tests for publication bias and discussed the likelihood and magnitude of impact of publication bias Yes No No meta-analysis conducted

Q16. Did the review authors report any potential sources of conflict of interest, including any funding they received for conducting the review? For Yes: The authors reported no competing interests OR The authors described their funding sources and how they managed potential conflicts of interest

**Supplementary Table 4:** Incidence of CVD after a cancer diagnosis

| Author | Population | Number of participants | Age | Population (cancer) | Population (CVD) | Comparator | Meta-analysis measure | Metrics of meta-analysis | outcome |
| --- | --- | --- | --- | --- | --- | --- | --- | --- | --- |
| Lun ^2^ | Adult | 2,552,121 |  | All cancers except non-melanoma skin cancers | Stroke |  | Random | Proportion | The cumulative incidence of stroke at 1 year was 1.4% (95% CI: 0.9–2.2%, I^2^ = 99.92%, p < 0.001).  Incidence of ischemic stroke 1.3% (95% CI: 1.0–1.8%, I^2^ = 99.76%, p < 0.001) and spontaneous intracerebral haemorrhage (ICH) 0.3% (95% CI: 0.1–0.9%, I^2^ = 99.4%, p < 0.001).  Stroke incidences at 1 month1.6% (95% CI: 0.2–10.8%; I^2^ = 99.78%, p <0.001), 3 month 1.0% (95% CI: 0.3– 2.8%; I^2^ = 99.95%, p <0.001), and 6 months 1.3% (95% CI: 0.6–2.9%; I^2^ = 99.96%, %, p <0.001).  In atrial fibrillation patients, the incidence of ischemic stroke was 3.3% at 1 year (95% CI: 2.4–4.6%, I^2^ = 29.70%, p = 0.24).  Stroke incidence in head and neck cancer 5.3% (95% CI: 0.8–27.5%, I^2^ = 99.83%, p = 0.00), hematologic cancer 3.9% (95% CI: 2.4–6.3%, I^2^ = 63.64%, p = 0.09), thyroid cancer 0.6% (95% CI: 0.1–4.8%, I^2^ = 96.13%, p = 0.00), breast cancer 0.4% (95% CI: 0.2–0.9%, I^2^ = 95.10%, p = 0.00), pancreatic 1.9% (95% CI: 1.6-2.2), cervical 2.1% (95% CI: 1.9-2.3), prostate 2.4% (95% CI: 2.3-2.5), colorectal 0.6% (95% CI: 0.5-0.6), hepatic 2.0% (95% CI: 1.8-2.2). |
| Horsted ^3^ | Adult | 490,720 |  | Breast, lung, colorectal, prostate, brain, bone, pancreatic, or haematologic | VTE | General population | Random | Incidence rate, incidence rate ratio | VTE incidence 43 per 1,000 person-years (95% CI: 26-72).  Risk of VTE (IRR = 3.96; 95% CI: 3.68-4.27) compared to the general population.    VTE incidence from cancer of brain 116(95% CI: 66-198), haematological 35(95% CI: 22-6), colorectal 33(95% CI: 21-53), lung 52(95% CI: 38-70), breast 21(95% CI: 10-41), bone 24(95% CI: 2-245), pancreatic 102(95% CI: 70-151), prostate 12(95% CI: 8-17). |
| Suleman ^4^ | Adult | 3,688 | 47 to 68 years | Primary central nervous system lymphoma | VTE |  | Random | Rate | Rate of VTE 11% (95% CI: 9%-14%, I^2^ = 81%; p < 0.001). |
| Caruso ^5^ | Adult | 18,018 | mean 62.4 | Lymphoma | VTE |  | Random | Rate | Rate of thrombosis 6.4% (95% CI: 6.0%-6.8%, p <o.o01). Rate of venous 5.3% (95% CI: 5.0%-5.7%), arterial events 1.1% (95% CI: 0.9%-1.2%).  VTE in non-Hodgkin lymphoma 6.5% (95% CI: 6.1%-6.9%), Hodgkin lymphoma 4.7% (95% CI: 3.9%-5.6%), CNS lymphoma 48.1% (95% CI: 32.8%–70.7%) |
| Malgor ^6^ | Adult | 114,922 | mean age 61 years, range 21 to 90 | Lung | Pulmonary embolism |  |  | Rate | Incidence of pulmonary embolism 3.6%, range 0.2% to 17%. |
| Yuan ^7^ | All | 5,889,234 | mean 53 to 75 | Solid cancer | Atrial fibrillation | Cancer free | Random | Odd ratio | Risk of atrial fibrillation (OR 1.47, 95% CI: 1.31-1.66, I^2^ = 67%, p < 0.00001) compared to noncancer patients.  The risk of atrial fibrillation within 90 days of cancer diagnosis (OR 7.62, 95% CI: 3.08- 18.88, p < 0.00001), 91 to 365 days (OR 1.06, 95% CI: 0.90-1.25, p = 0.46, I^2^ = 0%) or beyond 365 days (OR 0.97, 95% CI: 0.71-1.34, I^2^ = 84%, p = 0.87).  Risk of atrial fibrillation in colorectal cancer (OR 1.54: 95% CI: 1.40-1.71, I^2^ = 48%, p < 0.00001), breast cancer OR 2.07, 95% CI: 0.96- 4.45, I^2^ = 81%, p = 0.06 |
| Catueno ^8^ | Children |  | 30 months to 16 years | Cancer | Stroke |  |  | Rate | The incidence of stroke varied between 0.47% and 2.9%, |
| Weeks ^9^ | Adult | 6,324 | mean 60 years | Ovary | VTE | Cancer stage I/II | Random | Odds ratio | Risk of VTE in stage III/IV ovarian cancer 2.73 (95% CI: 1.84–4.06, I^2^ = 64%, p =0.01) compared to stage I/II |
| Bao ^11^ | Adult | 1,406,464 |  | Cancer | Atrial fibrillation | General population | Random | Rate | AF rate 0.7% (95% CI: 0.1–0.98%, I^2^ = 99.8%, p<0.001).  AF in breast cancer 0.9% (95% CI: 0.1–2.3%, I^2^ = 99.9%, p < 0.001). |
| Caruso ^13^ | Children | 1,752 | mean 5.5 years | Lymphoblastic leukaemia | Thrombosis |  |  | Rate | The rate of thrombosis 5.2% (95% CI: 4.2-6.4).  CNS 2.9% (95% CI: 2.2-3.8), non-CNS thrombosis events 2.3% (95% CI: 1.7-3.2). |
| Zhang ^14^ | All | 10,479,530 |  | Cancer | Stroke | Cancer-free population | Random | Relative risk | Risk of stroke 1.66 (95% CI: 1.35–2.04, I^2^ = 99.5%, p < 0.001) compared to cancer-free controls.  Risk of ischemic stroke (1.53, 95% CI: 1.28–1.84, I^2^ = 99.1%, p = 0.000), haemorrhagic stroke (1.83, 95% CI: 0.94–3.55, I^2^ = 97.4%).  Risk of stroke from haematological 1.42 (95% CI: 1.18–1.71, I^2^ = 77.9), head and neck 1.60 (95% CI: 1.12–2.29, I^2^ = 98.3), lung 1.57 (95% CI: 1.22–2.02, I^2^ = 95.7), stomach 1.11 (95% CI: 1.03–1.19), pancreatic 2.32 (95% CI: 1.86–2.88, I^2^ = 67.2), breast 1.10 (95% CI: 0.93-1.32, I^2^ = 93.1), colorectal 1.05 (95% CI: 0.99-1.12, I^2^ = 28.8), gynaecologic 1.14 (95% CI: 0.75-1.73, I^2^ = 96.3), melanoma 1.27 (95% CI: 0.92-1.75, I^2^ = 72.1), prostate 0.97 (95% CI: 0.88-1.06, I^2^ = 81.2), urinary tract 1.07 (95% CI: 0.98-1.18) |
| Balomenakis ^15^ | Adult | 3,149,547 |  | Cancer | Arterial thrombo-embolism | Cancer-free atrial fibrillation patients | Random | Odds ratio | Arterial thromboembolism 0.97 (95% CI: 0.85–1.11; I² = 87%). |
| Wu ^16^ | All | 162,126 |  | Leukaemia | VTE | General population | Random | Incidence rate | VTE in acute lymphoblastic leukemia (adult) 6% (95%CI: 2–12%, I^2^ = 60.5%, p = 0.019), acute lymphoblastic leukemia (children) 5% (95% CI: 3–7%, I^2^ = 66.2%, p = 0.00), chronic lymphoblastic leukemia 3% (95% CI: 2–5%, I^2^ = 0.0%, p = 0.74), acute myeloid leukemia 4% (95% CI: 2–7%, I^2^ = 0.0%, p = 0.90), Acute promyelocytic leukemia 4% (95%CI: 1–7%, I^2^ = 0.0%, p = 0.52), and chronic myeloid leukemia population 13% (95%CI: 1–36%, I^2^ = 48.2%, p = 0.00). |
| Kostopoulos ^17^ | All | 387,524 |  | Differentiated thyroid cancer | Atrial fibrillation | Cancer free | Random | Incidence rate, incidence rate ratio | Incidence rate of AF 4.86 (95% CI: 3.29-7.17, I^2^ = 96%, p = 0.20), incidence rate ratio 1.54 (95% CI: 1.44-1.65, I^2^ = 0%). |
| Monaghan ^18^ | Adult | 1,184,160 | Mean 58.4, range: 17–104 | Head and neck | Vascular events |  | Random | Proportion | Rate of pulmonary embolism 0.6% (95% CI: 0.4–0.8), myocardial infarction 2.0% (95% CI: 1.6-2.5), deep vein thrombosis 1.2% (95% CI: 0.9-1.5), cerebrovascular accident 2.7% (95% CI: 2.1-3.4), transient Ischemic attack 2.9 (95% CI: 1.9–4.3), Internal jugular vein thrombosis 12.1 (95% CI: 2.3–28.1), arterial thrombosis 1.9 (95% CI: 0.1–9.0). |
| Qian ^19^ |  |  |  | Brain | VTE | Cancer free | Random | Relative risk | Risk of VTE 1.66 (95% CI: 1.31–2.12, I^2^ = 63.7%, p = 0.001)  VTE risk with glioma (1.68, 95% CI: 1.44–1.98, I^2^ = 0.0% p = 0.001), high-grade glioma 1.70 (95% CI: 1.29–2.23, I^2^ = 0.0%, p = 0.001), glioblastoma multiforme 1.74 (95% CI: 1.43–2.12, I^2^ = 0.0%, p = 0.001) |
| Pastori ^20^ |  | 2,868,010 | mean 70.3 to 77.9 years | Cancer | Myocardial infarction, stroke | Cancer free | Random | Hazard ratio | Risk of intracranial haemorrhage 1.07 (95% CI: 1.04–1.11, I^2^ = 20%), stroke 0.91 (95% CI: 0.89–0.94, I^2^ = 97%), myocardial infarction 0.97 (95% CI: 0.94–1.01, I^2^ = 47%) |
| Wang ^22^ |  | 25,805 |  | Essential thrombo-cythemia | Thrombosis |  | Random | Rate | Incidence of arterial thrombosis 13.4% (95% CI: 12.0-14.8%, I^2^ = 93%), stroke 5.3% (95% CI: 4.0-6.7%, I^2  =^ 79%), transient ischemic attack 5.1% (95% CI: 3.3-7.8%, I^2^  = 90%), myocardial infarction 2.4% (95% CI: 1.9-3.0%, I^2^ = 59%), unstable angina 0.9% (95% CI: 0.5-1.2%, I^2^ = 0%), peripheral arterial thrombosis 2.0% (95% CI: 1.4-2.5%, I^2^ = 80%).  Incidence of venous thrombotic events 5.5% (95% CI: 4.7-6.2%, I^2^ = 88%), peripheral vein thrombosis 2.9% (95% CI: 2.3-3.6%, I^2^  = 0%), superficial vein thrombosis 1.8% (95% CI: 0.3-9.8%, I^2^ = 85%), deep vein thrombosis 1.6% (95% CI: 1.1-2.1%, I^2  =^ 57%), abdominal vein thrombosis 0.8% (95% CI: 0.4-1.2%, I^2^  = 62%), pulmonary embolism 0.3% (95% CI: 0.1-0.4%, I^2^ = 0%), cerebral vein thrombosis 0.2% (95% CI: 0-0.6%, I^2^ = 13%) |
| Mauro ^23^ | All | 166,271 | mean 56.88 years | Breast | Atrial fibrillation |  | Random | Proportion | Risk of atrial fibrillation 3% (95% CI: 2-5, I^2^ = 99.99%, p <0.01). AF in early 1% (95% CI: 0-3, I^2^ 99.69%, p <0.01), metastatic breast cancer 0% (95% CI: 0-1, I^2^ = 54.2%, p = 0.03) |
| Stone ^24^ | All | 32,438 | median age 27.1, range: 6 to 52 | Lymphoma | CVD | General population | Random | Standardised incidence ratio | Hodgkin Lymphoma  Risk of cerebrovascular disease 1.99 (95% CI: 1.56-2.55, I^2^ = 90.3%), cardiac dysrhythmia 3.23 (95% CI: 2.23-4.68, I^2^ = 93.0%), coronary heart disease 2.54 (95% CI: 1.95-3.31, I^2^ = 97.2%), CVD 2.37 (95% CI: 1.43-3.91, I^2^ = 98.1%), myocardial disease 3.95 (95% CI: 2.48-6.27, I^2^ = 97.1%, pericardial disease 10.67 (95% CI: 7.75-14.69, I^2^ = 63.3%), valvular heart disease 13.10 (95% CI: 7.41-23.16, I^2^ = 96.2%).  Non-Hodgkin's Lymphoma Risk of cerebrovascular disease 1.66 (95% CI: 1.46-1.88, I^2^ = 0.0%), cardiac dysrhythmia 1.96 (95% CI: 1.22-3.13, I^2^ = 72.3%), coronary heart disease 1.14 (95% CI: 0.95-1.37, I^2^ = 0.0%), CVD 1.90 (95% CI: 1.43-2.52, I^2^ = 86.7%), myocardial disease 5.38 (95% CI: 3.35-8.64, I^2^ = 89.8%), pericardial disease 4.70 (95% CI: 2.08-10.61),  valvular heart disease 3.76 (95% CI: 2.12-6.66, I^2^ = 1.5%) |
| Pergialiotis ^25^ | Adult | 5,414 |  | Ovary | Thrombosis | Ovary cancer patients without thrombosis | Random | Odds ratio | Incidence of deep vein thrombosis 1.62 (95 % CI: 0.48–5.46). |
| Govsyeyev ^27^ | Adult | 1,704 | 72± 3.9 | Cancer | Acute limb ischemia |  | Random | Percentage | 59% developed acute limb ischemia |
| Yang ^28^ | All | 9,000,000 |  | Lung | VTE, PE |  |  | Proportion | VTE 6% (95% CI: 5-8, I^2^ = 100%, p = 0.01), pulmonary embolism 3% (95% CI: 2-4, I^2^ = 100%, p = 0.01) |
| Wang ^29^ |  |  |  | Cancer | Acute myocardial infarction | Cancer-free acute MI patients | Random | OR | Risk of acute MI 1.16 (95% CI: 0.99-1.37, I^2^ = 89.3%, p <0.01) |
| Dongchen ^26^ | Adult | 7,210,530 |  | Cancer | CVD | Cancer-free myocardial infarction patients | Random | HR | Risk of CVD 1.26 (95% CI: 0.94–1.70, I^2^ = 99.2%, p=0.126), stroke 1.11 (95% CI: 0.97–1.27, I^2^ = 85.1%, p <0.05) |
| Veiga ^31^ | Adult |  |  | CNS malignant neoplasms | VTE |  | Random | Rate | VTE incidence 13.68% (95% CI: 9.79-18.79, I^2^ = 99%, p <0.01) |
| Betts ^33^ | All | 3,948,752 |  | Cancer | VTE |  | Random | Rate | 3.1% experienced VTE within 1 year of diagnosis  VTE risk in Brain 6.4% (95% CI: 4.4-9.2), colorectal 4.1%(95% CI: 3.1-5.5), gastrointestinal 4.9%(95% CI: 3.5-6.9), cervical 3.2%(95% CI: 1.9-5.2), uterine 3.1%(95% CI: 2.1-4.4), melanoma 0.7%(95% CI: 0.4-1.2), breast 1.8%(95% CI: 1.3-2.4), ovary 6.1%(95% CI: 4.3-8.5), pancreatic 7.4%(95% CI: 5.4-10.1), Hodkin lymphoma 4.4%(95% CI: 2-8.8), Non Hodgkin lymphoma 4.5%(95% CI: 3.1-6.6), renal 3.7%(95% CI: 2.4-5.8), bladder 3.1%(95% CI: 2.2-4.5), testicular 2.1%(95% CI: 1.2-3.7), oesophageal 3.7%(95% CI: 2.5-5.4), melanoma 4.9%(95% CI: 3.3-7.2), prostate 1.7%(95% CI: 1.2-2.4) |
| Harry ^34^ | Adult |  |  | Solid cancer | VTE |  |  | Percentage | Incidence of VTE was 9.74 %  VTE in brain 14.22 %, gastroesophageal 15.43 %, ovary 14.66%, prostate 1.57%, valvular 1.62% |
| Galimzhanov ov ^10^ | Adult | 2,111,882 | mean 46–76.8 | Breast | CAD, AF, HF, stroke | General cancer-free population | Random | Incidence rate per 1000-person year, Hazard ratio | IRs for HF 4.44 (95% CI: 3.33–5.92, I^2^ = 99.6%, p <0.01), CAD 4.29 (95% CI: 3.09–5.94, I^2^ = 99.7%, p <0.01), MI 1.98 (95% CI: 1.24– 3.16, I^2^ = 99.3%, p <0.01), stroke 4.33 (95% CI: 2.97–6.30, I^2^ = 99.6%, p <0.01), ischaemic stroke 2.64 (95% CI: 1.79–3.92, I^2^ = 96.9%, p <0.01), and AF 12.95 (95% CI: 12.60–13.31, I^2^ = 0.0%, p = 0.04). |

AF: Atrial fibrillation, CI: confidence interval, CVD: cardiovascular disease, HR: Hazard ratio, IR: incidence rate, IRR: incidence rate ratio, MI: myocardial infarction, OR: odds ratio, PE: pulmonary embolism, RR: risk ratio, VTE: venous thromboembolism

Supplementary Table 5. CVD incidence follow-up period and outcomes

| **Cancer type(s)** | **Cardiovascular outcome** | **Follow-up** | **Outcome measure** | **Results** |
| --- | --- | --- | --- | --- |
| Acute lymphoblastic leukaemia (adult) | VTE | NR | IR | 6 (95% CI: 2–12, I² = 60.5%, p = 0.019) |
| Acute lymphoblastic leukaemia (children) | VTE | NR | IR | 5 (95% CI: 3–7, I² = 66.2%, p = 0.00) |
| Acute myeloid leukaemia | VTE | NR | IR | 4 (95% CI: 2–7, I² = 0.0%, p = 0.90) |
| Acute promyelocytic leukaemia | VTE | NR | IR | 4 (95% CI: 1–7, I² = 0.0%, p = 0.52) |
| All cancer | Acute MI | NR | OR | 1.16 (95% CI: 0.99–1.37, I² = 89.3%, p < 0.01) |
| All cancer | Arterial thrombo-embolism | NR | OR | 0.97 (95% CI: 0.85–1.11; I² = 87%). |
| All cancer | AF | NR | Rate | 0.7% (95% CI: 0.1–0.98, I² = 99.8%, p < 0.001) |
| All cancer | CVD | NR | HR | 1.26 (95% CI: 0.94–1.70, I² = 99.2%, p = 0.126) |
| All cancer | Haemorrhagic stroke | NR | RR | 1.83 (95% CI: 0.94–3.55, I² = 97.4) |
| All cancer | Intracranial haemorrhage | NR | RR | 1.07 (95% CI: 1.04–1.11, I² = 20%) |
| All cancer | Ischaemic stroke | NR | RR | 1.53 (95% CI: 1.28–1.84, I² = 99.1%, p = 0.000) |
| All cancer | MI | NR | RR | 0.97 (95% CI: 0.94–1.01, I² = 47%) |
| All cancer | Stroke | NR | RR | 1.66 (95% CI: 1.35–2.04, I² = 99.5%, p < 0.001) |
| All cancer | Stroke | NR | RR | 0.91 (95% CI: 0.89–0.94, I² = 97%) |
| All cancer | Stroke | NR | HR | 1.11 (95% CI: 0.97–1.27, I² = 85.1%, p < 0.05) |
| All cancers except non-melanoma skin cancers | Intracerebral haemorrhage | 1 year | Proportion | 0.3% (95% CI: 0.1–0.9, I^2^ = 99.4%, p < 0.001) |
| All cancers except non-melanoma skin cancers | Ischemic stroke | 1 year | Proportion | 1.3% (95% CI: 1.0–1.8, I^2^ = 99.76%, p < 0.001) |
| All cancers except non-melanoma skin cancers | Ischemic stroke in patients with atrial fibrillation | 1 year | Proportion | 3.3% (95% CI: 2.4–4.6, I^2^ = 29.70%, p = 0.24) |
| All cancers except non-melanoma skin cancers | Stroke | 1 year | Proportion | 1.4% (95% CI: 0.9–2.2, I^2^ = 99.92%, p < 0.001). |
| All cancers except non-melanoma skin cancers | Stroke | 1 month | Proportion | 1.6% (95% CI: 0.2–10.8; I^2^ = 99.78%, p <0.001) |
| All cancers except non-melanoma skin cancers | Stroke | 3 months | Proportion | 1.0% (95% CI: 0.3– 2.8; I^2^ = 99.95%, p <0.001) |
| All cancers except non-melanoma skin cancers | Stroke | 6 months | Proportion | 1.3% (95% CI: 0.6–2.9; I^2^ = 99.96%, %, p <0.001) |
| Bladder cancer | VTE | NR | Rate | 3.1% (95% CI: 2.2–4.5) |
| Bone cancer | VTE | NR | IRR | 24 (95% CI: 2–245) |
| Brain cancer | VTE | NR | IRR | 116 (95% CI: 66–198) |
| Brain cancer | VTE | NR | RR | 1.66 (95% CI: 1.31–2.12, I² = 63.7%, p = 0.001) |
| Brain cancer | VTE | NR | Rate | 6.4% (95% CI: 4.4–9.2) |
| Breast cancer | AF | NR | OR | 2.07 (95% CI: 0.96–4.45, I² = 81%, p = 0.06) |
| Breast cancer | AF | NR | Rate | 0.9% (95% CI: 0.1–2.3, I² = 99.9%, p < 0.001) |
| Breast cancer | AF | NR | Proportion | 3% (95% CI: 2–5, I² = 99.99%, p < 0.01) |
| Breast cancer | AF | NR | IR | 12.95 (95% CI: 12.60–13.31, I² = 0%, p = 0.04) |
| Breast cancer | Coronary artery disease | NR | IR | 4.29 (95% CI: 3.09–5.94, I² = 99.7%, p < 0.01) |
| Breast cancer | Heart failure | NR | IR | 4.44 (95% CI: 3.33–5.92, I² = 99.6%, p < 0.01) |
| Breast cancer | Ischaemic stroke | NR | IR | 2.64 (95% CI: 1.79–3.92, I² = 96.9%, p < 0.01) |
| Breast cancer | MI | NR | IR | 1.98 (95% CI: 1.24–3.16, I² = 99.3%, p < 0.01) |
| Breast cancer | Stroke | 1 year | Proportion | 0.4% (95% CI: 0.2–0.9, I2 = 95.10%, p = 0.00) |
| Breast cancer | Stroke | NR | RR | 1.10 (95% CI: 0.93–1.32, I² = 93.1) |
| Breast cancer | Stroke | NR | IR | 4.33 (95% CI: 2.97–6.30, I² = 99.6%, p < 0.01) |
| Breast cancer | VTE | NR | IRR | 21 (95% CI: 10–41) |
| Breast cancer | VTE | NR | Rate | 1.8% (95% CI: 1.3–2.4) |
| Breast cancer (early) | AF | NR | Proportion | 1% (95% CI: 0–3, I² = 99.69%, p < 0.01) |
| Breast cancer (metastatic) | AF | NR | Proportion | 0% (95% CI: 0–1, I² = 54.2%, p = 0.03) |
| Breast, lung, colorectal, prostate, brain, bone, pancreatic, or haematologic cancers | VTE | NR | IR | 43 (95% CI: 26–72) |
| Breast, lung, colorectal, prostate, brain, bone, pancreatic, or haematologic cancers | VTE | NR | IRR | 3.96 (95% CI: 3.68–4.27) |
| Cervical cancer | Stroke | 1 year | Proportion | 2.1% (95% CI: 1.9–2.3%) |
| Cervical cancer | VTE | NR | Rate | 3.2% (95% CI: 1.9–5.2) |
| Chronic lymphoblastic leukaemia | VTE | NR | IR | 3 (95% CI: 2–5, I² = 0.0%, p = 0.74) |
| Chronic myeloid leukaemia | VTE | NR | IR | 13 (95% CI: 1–36, I² = 48.2%, p = 0.00) |
| CNS malignant neoplasms | VTE | NR | Rate | 13.68% (95% CI: 9.79–18.79, I² = 99%, p < 0.01) |
| Colorectal cancer | AF | NR | OR | 1.54 (95% CI: 1.40–1.71, I² = 48%, p < 0.001) |
| Colorectal cancer | Stroke | 1 year | Proportion | 0.6% (95% CI: 0.5–0.6%) |
| Colorectal cancer | Stroke | NR | RR | 1.05 (95% CI: 0.99–1.12, I² = 28.8) |
| Colorectal cancer | VTE | NR | IRR | 33 (95% CI: 21–53) |
| Colorectal cancer | VTE | NR | Rate | 4.1% (95% CI: 3.1–5.5) |
| Differentiated thyroid cancer | AF | 4.3-18.8 years | IR | 4.86 (95% CI: 3.29–7.17, I² = 96%, p = 0.20) |
| Differentiated thyroid cancer | AF | 4.3-18.8 years | IRR | 1.54 (95% CI: 1.44–1.65, I² = 0%) |
| Essential thrombocythaemia | Abdominal vein thrombosis | NR | Rate | 0.8% (95% CI: 0.4–1.2, I² = 62%) |
| Essential thrombocythaemia | Arterial thrombosis | NR | HR | 13.4 (95% CI: 12.0–14.8%, I² = 93%) |
| Essential thrombocythaemia | Cerebral vein thrombosis | NR | Rate | 0.2% (95% CI: 0–0.6, I² = 13%) |
| Essential thrombocythaemia | Deep vein thrombosis | NR | Rate | 1.6% (95% CI: 1.1–2.1, I² = 57%) |
| Essential thrombocythaemia | MI | NR | HR | 2.4 (95% CI: 1.9–3.0%, I² = 59%) |
| Essential thrombocythaemia | Peripheral arterial thrombosis | NR | Rate | 2.0% (95% CI: 1.4–2.5, I² = 80%) |
| Essential thrombocythaemia | Peripheral vein thrombosis | NR | Rate | 2.9% (95% CI: 2.3–3.6, I² = 0%) |
| Essential thrombocythaemia | PE | NR | Rate | 0.3% (95% CI: 0.1–0.4, I² = 0%) |
| Essential thrombocythaemia | Stroke | NR | HR | 5.3 (95% CI: 4.0–6.7%, I² = 79%) |
| Essential thrombocythaemia | Superficial vein thrombosis | NR | Rate | 1.8% (95% CI: 0.3–9.8, I² = 85%) |
| Essential thrombocythaemia | Transient ischaemic attack | NR | HR | 5.1 (95% CI: 3.3–7.8%, I² = 90%) |
| Essential thrombocythaemia | Unstable angina | NR | Rate | 0.9% (95% CI: 0.5–1.2, I² = 0%) |
| Essential thrombocythaemia | VTE | NR | Rate | 5.5% (95% CI: 4.7–6.2, I² = 88%) |
| Gastrointestinal cancer | VTE | NR | Rate | 4.9% (95% CI: 3.5–6.9) |
| Glioblastoma multiforme | VTE | NR | RR | 1.74 (95% CI: 1.43–2.12, I² = 0%, p = 0.001) |
| Glioma | VTE | NR | RR | 1.68 (95% CI: 1.44–1.98, I² = 0%, p = 0.001) |
| Gynaecologic cancer | Stroke | NR | RR | 1.14 (95% CI: 0.75–1.73, I² = 96.3) |
| Haematologic cancer | Stroke | 1 year | Proportion | 3.9% (95% CI: 2.4–6.3, I^2^ = 63.64%, p = 0.09) |
| Haematologic cancer | VTE | NR | IRR | 35 (95% CI: 22–56) |
| Haematological cancer | Stroke | NR | RR | 1.42 (95% CI: 1.18–1.71, I² = 77.9) |
| Head and neck cancer | Arterial thrombosis | NR | Proportion | 1.9% (95% CI: 0.1–9.0) |
| Head and neck cancer | Cerebrovascular accident | NR | Proportion | 2.7% (95% CI: 2.1–3.4) |
| Head and neck cancer | Deep vein thrombosis | NR | Proportion | 1.2% (95% CI: 0.9–1.5) |
| Head and neck cancer | Internal jugular vein thrombosis | NR | Proportion | 12.1% (95% CI: 2.3–28.1) |
| Head and neck cancer | MI | NR | Proportion | 2.0% (95% CI: 1.6–2.5) |
| Head and neck cancer | PE | NR | Proportion | 0.6% (95% CI: 0.4–0.8) |
| Head and neck cancer | Stroke | 1 year | Proportion | 5.3% (95% CI: 0.8–27.5, I^2^ = 99.83%, p = 0.00) |
| Head and neck cancer | Stroke | NR | RR | 1.60 (95% CI: 1.12–2.29, I² = 98.3) |
| Head and neck cancer | Transient ischaemic attack | NR | Proportion | 2.9% (95% CI: 1.9–4.3) |
| Hepatic cancer | Stroke | 1 year | Proportion | 2.0% (95% CI: 1.8–2.2%) |
| High-grade glioma | VTE | NR | RR | 1.70 (95% CI: 1.29–2.23, I² = 0%, p = 0.001) |
| Hodgkin lymphoma | Cardiac dysrhythmia | ≥5 years | SIR | 3.23 (95% CI: 2.23–4.68, I² = 93.0%) |
| Hodgkin lymphoma | Coronary heart disease | ≥5 years | SIR | 2.54 (95% CI: 1.95–3.31, I² = 97.2%) |
| Hodgkin lymphoma | CVD | ≥5 years | SIR | 1.99 (95% CI: 1.56–2.55, I² = 90.3%) |
| Hodgkin lymphoma | CVD | ≥5 years | SIR | 2.37 (95% CI: 1.43–3.91, I² = 98.1%) |
| Hodgkin lymphoma | Myocardial disease | ≥5 years | SIR | 3.95 (95% CI: 2.48–6.27, I² = 97.1%) |
| Hodgkin lymphoma | Pericardial disease | ≥5 years | SIR | 10.67 (95% CI: 7.75–14.69, I² = 63.3%) |
| Hodgkin lymphoma | Valvular heart disease | ≥5 years | SIR | 13.10 (95% CI: 7.41–23.16, I² = 96.2%) |
| Hodgkin lymphoma | VTE | NR | Rate | 4.4% (95% CI: 2–8.8) |
| Lung cancer | PE | NR | Proportion | 3% (95% CI: 2–4, I² = 100%, p = 0.01) |
| Lung cancer | Stroke | NR | RR | 1.57 (95% CI: 1.22–2.02, I² = 95.7) |
| Lung cancer | VTE | NR | IRR | 52 (95% CI: 38–70) |
| Lung cancer | VTE | NR | Proportion | 6% (95% CI: 5–8, I² = 100%, p = 0.01) |
| Lymphoblastic leukaemia | CNS thrombosis | NR | Rate | 2.9% (95% CI: 2.2–3.8) |
| Lymphoblastic leukaemia | Non-CNS thrombosis | NR | Rate | 2.3% (95% CI: 1.7–3.2) |
| Lymphoblastic leukaemia | Thrombosis | NR | Rate | 5.2% (95% CI: 4.2–6.4) |
| Lymphoma | Thrombosis | NR | Proportion | 6.4% (95% CI: 6.0-6.8, p <0.001). |
| Lymphoma | VTE | NR | Proportion | 5.3% (95% CI: 5.0-5.7) |
| Lymphoma (CNS) | VTE | NR | Proportion | 11% (95% CI: 9-14, I^2^ = 81%; p < 0.001) |
| Lymphoma (CNS) | VTE | NR | Proportion | 48.1% (95% CI: 32.8–70.7) |
| Lymphoma (Hodgkin) | VTE | NR | Proportion | 4.7% (95% CI: 3.9-5.6), |
| Lymphoma (Non-Hodgkin) | VTE | NR | Proportion | 6.5% (95% CI: 6.1-6.9), |
| Lymphoma (Non-Hodgkin) | Cardiac dysrhythmia | ≥5 years | SIR | 1.96 (95% CI: 1.22–3.13, I² = 72.3%) |
| Lymphoma (Non-Hodgkin) | Coronary heart disease | ≥5 years | SIR | 1.14 (95% CI: 0.95–1.37, I² = 0%) |
| Lymphoma (Non-Hodgkin) | CVD | ≥5 years | SIR | 1.66 (95% CI: 1.46–1.88, I² = 0%) |
| Lymphoma (Non-Hodgkin) | CVD | ≥5 years | SIR | 1.90 (95% CI: 1.43–2.52, I² = 86.7%) |
| Lymphoma (Non-Hodgkin) | Myocardial disease | ≥5 years | SIR | 5.38 (95% CI: 3.35–8.64, I² = 89.8%) |
| Lymphoma (Non-Hodgkin) | Pericardial disease | ≥5 years | SIR | 4.70 (95% CI: 2.08–10.61) |
| Lymphoma (Non-Hodgkin) | Valvular heart disease | ≥5 years | SIR | 3.76 (95% CI: 2.12–6.66, I² = 1.5%) |
| Lymphoma (Non-Hodgkin) | VTE | NR | Rate | 4.5% (95% CI: 3.1–6.6) |
| Melanoma | Stroke | NR | RR | 1.27 (95% CI: 0.92–1.75, I² = 72.1) |
| Melanoma | VTE | NR | Rate | 0.7% (95% CI: 0.4–1.2) |
| Oesophageal cancer | VTE | NR | Rate | 3.7% (95% CI: 2.5–5.4) |
| Ovary | Deep vein thrombosis | NR | OR | 1.62 (95% CI: 0.48–5.46) |
| Ovary | VTE | NR | OR | 2.73 (95% CI: 1.84–4.06, I² = 64%, p = 0.01) |
| Ovary cancer | VTE | NR | Rate | 6.1% (95% CI: 4.3–8.5) |
| Pancreatic cancer | Stroke | 1 year | Proportion | 1.9% (95% CI: 1.6-2.2) |
| Pancreatic cancer | Stroke | NR | RR | 2.32 (95% CI: 1.86–2.88, I² = 67.2) |
| Pancreatic cancer | VTE | NR | IRR | 102 (95% CI: 70–151) |
| Pancreatic cancer | VTE | NR | Rate | 7.4% (95% CI: 5.4–10.1) |
| Prostate cancer | Stroke | 1 year | Proportion | 2.4% (95% CI: 2.3–2.5%) |
| Prostate cancer | Stroke | NR | RR | 0.97 (95% CI: 0.88–1.06, I² = 81.2) |
| Prostate cancer | VTE | NR | IRR | 12 (95% CI: 8–17) |
| Prostate cancer | VTE | NR | Rate | 1.7% (95% CI: 1.2–2.4) |
| Renal cancer | VTE | NR | Rate | 3.7% (95% CI: 2.4–5.8) |
| Solid cancer | AF | NR | OR | 1.47 (95% CI: 1.31–1.66, I² = 67%, p < 0.001) |
| Solid cancer | AF | ≤90 days | OR | 7.62 (95% CI: 3.08–18.88, p < 0.001) |
| Solid cancer | AF | 91–365 days | OR | 1.06 (95% CI: 0.90–1.25, I² = 0%, p = 0.46) |
| Solid cancer | AF | >365 days | OR | 0.97 (95% CI: 0.71–1.34, I² = 84%, p = 0.87) |
| Stomach cancer | Stroke | NR | RR | 1.11 (95% CI: 1.03–1.19) |
| Testicular cancer | VTE | NR | Rate | 2.1% (95% CI: 1.2–3.7) |
| Thyroid cancer | Stroke | 1 year | Proportion | 0.6% (95% CI: 0.1–4.8, I^2^ = 96.13%, p = 0.00) |
| Urinary tract cancer | Stroke | NR | RR | 1.07 (95% CI: 0.98–1.18) |
| Uterine cancer | VTE | NR | Rate | 3.1% (95% CI: 2.1–4.4) |

AF: Atrial fibrillation, CI: confidence interval, CVD: cardiovascular disease, CNS: central nervous, system, HR: Hazard ratio, IR: incidence rate, IRR: incidence rate ratio, MI: myocardial infarction, OR: odds ratio, PE: pulmonary embolism, RR: risk ratio, VTE: venous thromboembolism

**Supplementary Table 6.** CVD-related mortality after a cancer diagnosis

| Author | Population | Number of participants | Age | Patient population (cancer) | Patient population (CVD) | Comparator | Meta-analysis measure | Outcome metrics | Outcome |
| --- | --- | --- | --- | --- | --- | --- | --- | --- | --- |
| Shin Ng ^1^ | Adult | 157 to 7,529,481 | 15-39 years | Cancer | CVD | General population | Random | standardised mortality ratios (SMR) | Risk of all CVD deaths from all cancers (1.55, 95% CI: 1.40–1.72, p <0.001)  Risk of death from diseases of heart/ ischaemic heart disease from all cancer (1.36, 95% CI: 1.29–1.44, p <0.001).  Risk of death from cerebrovascular disease from all cancer (1.56, 95% CI: 1.39–1.76, p <0.001)  All CVD death from cancer of Brain and other CNS 2.82 (95% CI: 2.20-3.63), haematological 2.42 (95% CI: 2.23-2.63), head and neck 1.81 (95% CI: 1.57-2.08), gastrointestinal 1.73 (95% CI: 1.34-2.24), lung 1.72 (95% CI: 1.42-2.08), urological 1.53 (95% CI: 1.22-1.90), gynaecological 1.41 (95% CI: 1.12-1.76), sarcoma 1.28 (95% CI: 1.14-1.43), breast 1.23 (95% CI: 1.03-1.47), skin and melanoma 1.20 (95% CI: 1.06-1.35), testicular and other germ cell tumours 1.14 (95% CI: 1.04-1.25), thyroid 1.10 (95% CI: 0.92-1.32), prostate 0.93 (95% CI: 0.86-1.00).  Risk of death from diseases of heart/heart disease/ischemic heart disease death from Brain and other CNS 1.66 (95% CI: 1.22-2.27), haematological 2.46 (95% CI: 2.19-2.77), head and neck 1.63 (95% CI: 1.22-2.18), gastrointestinal 1.34 (95% CI: 1.14-1.58), lung 1.53 (95% CI: 0.92-2.56), urological 1.57 (1.4-1.77), gynaecological 1.13 (95% CI: 0.91-1.41), sarcoma 1.24 (95% CI: 0.99-1.55), breast 1.14 (95% CI: 0.98-1.32), skin and melanoma 1.14 (95% CI: 0.89-1.46), testicular and other germ cell tumours 1.23 (95% CI: 0.98-1.55), thyroid 0.72 (95% CI: 0.47-1.09), prostate 0.82 (95% CI: 0.70-0.96).  Risk of death from cerebrovascular disease from brain and other CNS 5.95 (95% CI: 3.96-9.0), haematological 1.77 (95% CI: 1.54-2.04), head and neck 1.81 (95% CI: 1.33-2.47), gastrointestinal 2.31 (95% CI: 1.35-3.95), lung 1.83 (95% CI: 1.17-2.86), urological 1.25 (95% CI: 1.12-1.39), gynaecological 1.27 (95% CI: 0.99-1.64), sarcoma 0.97 (95% CI: 0.72-1.31), breast 0.97 (95% CI: 0.72-1.31), skin and melanoma 1.31 (95% CI: 1.09-1.58), testicular and other germ cell tumours 1.07 (95% CI: 0.65-1.76), thyroid 1.25 (95% CI: 0.7-2.23), prostate 0.93 (95% CI: 0.75-1.16). |
| Galimzhanov ^10^ | Adult | 2,111,882 | mean 46–76.8 | Breast | CVD, CAD, AF, HF, stroke | General cancer-free population | Random | Incidence rate per 1000-person year, Hazard ratio | IRs for cardiovascular death 1.73 (95% CI: 1.18–2.53, I^2^ = 99.8%, p <0.01).  Death from CVD (HR 1.09, 95% CI: 1.07–1.11, I^2^ = 0.0%, p =0.31) during the first 5 years following BC diagnosis. Between 8 and 11 years following BC diagnosis (HR 1.23, 95% CI: 0.99–1.52, I^2^ = 93.7%, p <0.01), stroke during 8 years from the index date (HR 0.99, 95% CI: 0.83–1.19, I^2^ = 93.6%, p <0.01; ischaemic stroke (HR 1.19, 95% CI: 0.94–1.51, I^2^ = 69.7%, p =0.03), AF up to 3 months: HR 1.64, 95% CI: 1.18–2.26, I^2^ = 95.4%, p <0.01; from 3 months to 3 years: HR 1.13, 95% CI: 1.05–1.21, I^2^ =38.3%, p =0.19, CAD to 5 years (HR 0.97, 95% CI: 0.90–1.02, I^2^ = 73.9%, p <0.01) and from 5 to 8 years of follow-up HR 1.01, 95% CI: 0.92–1.10, I^2^ = 82.4%, p<0.01, HF to first year (HR 1.29, (95% CI: 1.3-1.63, I^2^ = 97.3%, p <0.01) from 1 to 2 years (HR 1.21, 95% CI: 1.1–1.33, I^2^ = 82.8%, p <0.01), 2 to 5 years (HR 1.22, 95% CI: 1.11–1.33, I^2^ = 83.1%, p <0.01), and 5 to 10 years (HR 1.19, 95% CI: 1.1–1.29, I^2^ = 73.0%, p <0.01). |
| Boyne ^12^ | All | 61,593 | median 25.8, range, 6.6-56.8 | Lymphoma | CVD | General | Random | Standardised mortality ratio | CVD death from Hodgkin 7.31 (95% CI: 5.29-10.10, I^2^ = 95.4%, p = 0.00) and non-Hodgkin 5.35 (95% CI: 2.55-11.24, I^2^ = 94.0%, p = 0.00) |
| Pastori ^20^ | Adult | 2,868,010 | mean 70.3 to 77.9 years | Cancer | Stroke, MI, CVD | Cancer free | Random | Hazard ratio | CVD death 1.01 (95% CI: 0.99–1.03, I^2^ = 87%) |
| Khan ^21^ | Adult | 13,202 |  | Breast | VTE | Breast cancer patients without VTE | Random | Hazard ratio | VTE death 1.70 (95% CI: 1.07– 2.71, I^2^ = 77.4%) |
| Dongchen ^26^ | Adult | 7,210,530 |  | Cancer | CVD | Cancer-free MI patients | Random | Hazard ratio | Cardiovascular mortality (HR 1.18, 95% CI: 0.91–1.54, I^2^ = 52.4%, p=0.050) compared to cancer-free MI patient. Mortality during hospitalization (0.69, 95% CI: 0.39–1.23, I^2^ = 0%, p=0.866) or in follow-up years (1.31, 95% CI: 0.97–1.78, I^2^ = 62.5%, p=0.030). |
| Wang ^29^ |  |  |  | Cancer | Acute MI | Cancer-free AMI patients | Random | Odd ratio and Hazard ratio | Death from cardiovascular complications (OR 2.06, 95% CI: 1.17-3.65, I^2^ = 55.4%, p=0.08), or (HR 1.20, 95% CI: 0.96-1.50; I^2^ = 74.4%, p <0.001) |
| Suleman ^4^ | Adult | 3,688 | 47 to 68 years | Primary CNS lymphoma | VTE |  | Random | Rate | Seventeen of the thrombotic events were fatal PEs (4.05%). |
| Route ^32^ | All |  |  | Cancer | Acute coronary syndrome | Cancer free | Random | Relative risk | In-hospital cardiac death (2.44, 95% CI: 1.73-3.44, I^2^ = 0%, p = 0.76) and one-year cardiac death (1.89, 95% CI: 1.25-2.86, I^2^ = 0%, p = 0.43) |
| Hua ^30^ | Adult | 15,305 |  | Lung | Pulmonary embolism | Lung cancer patients | Fixed | Odds ratio | 0.32 (95% CI: 0.21-0.49, I^2^ = 30%, p < 0.01) |

AF: Atrial fibrillation, CAD: coronary artery disease, CNS: central nervous system, CI: confidence interval, CVD: cardiovascular disease, HF: heart failure, HR: Hazard ratio, IR: incidence rate, IRR: incidence rate ratio, MI: myocardial infarction, OR: odds ratio, PE: pulmonary embolism, RR: risk ratio, SMR: standardised mortality ratio, VTE: venous thromboembolism

Supplementary Table 7. CVD mortality follow-up period and outcome

| **Cancer type(s)** | **Cardiovascular outcome** | **Follow-up** | **Outcome measure** | **Results** |
| --- | --- | --- | --- | --- |
| All cancer | Acute coronary syndrome | In-hospital | RR | 2.44 (95% CI: 1.73-3.44, I^2^ = 0%, p = 0.76) |
| All cancer | Acute coronary syndrome | 1 year | RR | 1.89 (95% CI: 1.25-2.86, I^2^ = 0%, p = 0.43) |
| All cancer | Acute MI | Hospital | OR | 2.06 (95% CI: 1.17–3.65) |
| All cancer | Acute MI | NR | HR | 1.20 (95% CI: 0.96–1.50) |
| All cancer | CVD | NR | SMR | 1.55 (95% CI: 1.40–1.72, p < 0.001) |
| All cancer | CVD | NR | SMR | 1.56 (95% CI: 1.39–1.76, p < 0.001) |
| All cancer | CVD | NR | HR | 1.01 (95% CI: 0.99–1.03) |
| All cancer | CVD | Hospital + follow-up | HR | 1.18 (95% CI: 0.91–1.54) |
| All cancer | Ischaemic heart disease | NR | SMR | 1.36 (95% CI: 1.29–1.44, p < 0.001) |
| Brain & CNS cancer | CVD | NR | SMR | 2.82 (95% CI: 2.20–3.63) |
| Brain & CNS cancer | CVD | NR | SMR | 5.95 (95% CI: 3.96–9.0) |
| Brain & CNS cancer | Heart disease | NR | SMR | 1.66 (95% CI: 1.22–2.27) |
| Breast cancer | AF | ≤3 months | HR | 1.64 (95% CI: 1.18–2.26) |
| Breast cancer | AF | 3 months–3 years | HR | 1.13 (95% CI: 1.05–1.21) |
| Breast cancer | CAD | ≤5 years | HR | 0.97 (95% CI: 0.90–1.02) |
| Breast cancer | CAD | 5–8 years | HR | 1.01 (95% CI: 0.92–1.10) |
| Breast cancer | CVD | NR | SMR | 1.23 (95% CI: 1.03–1.47) |
| Breast cancer | CVD | NR | SMR | 0.97 (95% CI: 0.72–1.31) |
| Breast cancer | CVD | First 5 years | HR | 1.09 (95% CI: 1.07–1.11) |
| Breast cancer | CVD | 8–11 years | HR | 1.23 (95% CI: 0.99–1.52) |
| Breast cancer | Heart disease | NR | SMR | 1.14 (95% CI: 0.98–1.32) |
| Breast cancer | HF | ≤1 year | HR | 1.29 (95% CI: ~1.3–1.63) |
| Breast cancer | HF | 1–2 years | HR | 1.21 (95% CI: 1.10–1.33) |
| Breast cancer | HF | 2–5 years | HR | 1.22 (95% CI: 1.11–1.33) |
| Breast cancer | HF | 5–10 years | HR | 1.19 (95% CI: 1.10–1.29) |
| Breast cancer | Stroke | 8 years | HR | 0.99 (95% CI: 0.83–1.19) |
| Breast cancer | VTE | NR | HR | 1.70 (95% CI: 1.07–2.71) |
| Gastrointestinal cancer | CVD | NR | SMR | 1.73 (95% CI: 1.34–2.24) |
| Gastrointestinal cancer | CVD | NR | SMR | 2.31 (95% CI: 1.35–3.95) |
| Gastrointestinal cancer | Heart disease | NR | SMR | 1.34 (95% CI: 1.14–1.58) |
| Gynaecological cancer | CVD | NR | SMR | 1.41 (95% CI: 1.12–1.76) |
| Gynaecological cancer | CVD | NR | SMR | 1.27 (95% CI: 0.99–1.64) |
| Gynaecological cancer | Heart disease | NR | SMR | 1.13 (95% CI: 0.91–1.41) |
| Haematological cancer | CVD | NR | SMR | 2.42 (95% CI: 2.23–2.63) |
| Haematological cancer | CVD | NR | SMR | 1.77 (95% CI: 1.54–2.04) |
| Haematological cancer | Heart disease | NR | SMR | 2.46 (95% CI: 2.19–2.77) |
| Head & neck cancer | CVD | NR | SMR | 1.81 (95% CI: 1.57–2.08) |
| Head & neck cancer | CVD | NR | SMR | 1.81 (95% CI: 1.33–2.47) |
| Head & neck cancer | Heart disease | NR | SMR | 1.63 (95% CI: 1.22–2.18) |
| Lung cancer | CVD | NR | SMR | 1.72 (95% CI: 1.42–2.08) |
| Lung cancer | CVD | NR | SMR | 1.83 (95% CI: 1.17–2.86) |
| Lung cancer | Heart disease | NR | SMR | 1.53 (95% CI: 0.92–2.56) |
| Lung cancer | PE | NR | OR | 0.32 (95% CI: 0.21–0.49) |
| Lymphoma (Hodgkin) | CVD | >5 years | SMR | 7.31 (95% CI: 5.29–10.10) |
| Lymphoma (Non-Hodgkin) | CVD | >5 years | SMR | 5.35 (95% CI: 2.55–11.24) |
| Prostate cancer | CVD | NR | SMR | 0.93 (95% CI: 0.86–1.00) |
| Prostate cancer | CVD | NR | SMR | 0.93 (95% CI: 0.75–1.16) |
| Prostate cancer | Heart disease | NR | SMR | 0.82 (95% CI: 0.70–0.96) |
| Sarcoma | CVD | NR | SMR | 1.28 (95% CI: 1.14–1.43) |
| Sarcoma | CVD | NR | SMR | 0.97 (95% CI: 0.72–1.31) |
| Sarcoma | Heart disease | NR | SMR | 1.24 (95% CI: 0.99–1.55) |
| Skin/melanoma | CVD | NR | SMR | 1.20 (95% CI: 1.06–1.35) |
| Skin/melanoma | CVD | NR | SMR | 1.31 (95% CI: 1.09–1.58) |
| Skin/melanoma | Heart disease | NR | SMR | 1.14 (95% CI: 0.89–1.46) |
| Testicular/germ cell | CVD | NR | SMR | 1.14 (95% CI: 1.04–1.25) |
| Testicular/germ cell | CVD | NR | SMR | 1.07 (95% CI: 0.65–1.76) |
| Testicular/germ cell | Heart disease | NR | SMR | 1.23 (95% CI: 0.98–1.55) |
| Thyroid cancer | CVD | NR | SMR | 1.10 (95% CI: 0.92–1.32) |
| Thyroid cancer | CVD | NR | SMR | 1.25 (95% CI: 0.70–2.23) |
| Thyroid cancer | Heart disease | NR | SMR | 0.72 (95% CI: 0.47–1.09) |
| Urological cancer | CVD | NR | SMR | 1.53 (95% CI: 1.22–1.90) |
| Urological cancer | CVD | NR | SMR | 1.25 (95% CI: 1.12–1.39) |
| Urological cancer | Heart disease | NR | SMR | 1.57 (95% CI: 1.40–1.77) |

AF: Atrial fibrillation, CNS: central nervous system, CI: confidence interval, CVD: cardiovascular disease, HF: heart failure, HR: Hazard ratio, IR: incidence rate, IRR: incidence rate ratio, MI: myocardial infarction, OR: odds ratio, PE: pulmonary embolism, RR: risk ratio, SMR: standardised mortality ratio, VTE: venous thromboembolism

1.	Ng HS, Meng R, Marin TS, et al. Cardiovascular mortality in people with cancer compared to the general population: A systematic review and meta‐analysis. *Cancer Medicine*. 2024;13(15):e70057.

2.	Lun R, Roy DC, Hao Y, et al. Incidence of stroke in the first year after diagnosis of cancer—A systematic review and meta-analysis. *Frontiers in Neurology*. 2022;13:966190.

3.	Horsted F, West J, Grainge MJ. Risk of venous thromboembolism in patients with cancer: a systematic review and meta-analysis. 2012;

4.	Suleman A, Wine R, Carrier M, Hicks LK. The Risk of Venous Thromboembolism in Primary Central Nervous System Lymphoma: A Systematic Review and Meta Analysis. *Research and Practice in Thrombosis and Haemostasis*. 2024:102507.

5.	Caruso V, Di Castelnuovo A, Meschengieser S, et al. Thrombotic complications in adult patients with lymphoma: a meta-analysis of 29 independent cohorts including 18 018 patients and 1149 events. *Blood, The Journal of the American Society of Hematology*. 2010;115(26):5322-5328.

6.	Malgor RD, Bilfinger TV, Labropoulos N. A systematic review of pulmonary embolism in patients with lung cancer. *The Annals of thoracic surgery*. 2012;94(1):311-316.

7.	Yuan M, Zhang Z, Tse G, et al. Association of cancer and the risk of developing atrial fibrillation: a systematic review and meta‐analysis. *Cardiology Research and Practice*. 2019;2019(1):8985273.

8.	Catueno S, Ali S, Barrientos R, et al. Stroke in pediatric cancer patients: a systematic review. *Journal of Pediatric Hematology/Oncology*. 2023;45(6):291-300.

9.	Weeks KS, Herbach E, McDonald M, Charlton M, Schweizer ML. Meta‐analysis of VTE risk: ovarian cancer patients by stage, histology, cytoreduction, and ascites at diagnosis. *Obstetrics and gynecology international*. 2020;2020(1):2374716.

10.	Galimzhanov A, Istanbuly S, Tun HN, et al. Cardiovascular outcomes in breast cancer survivors: a systematic review and meta-analysis. *European Journal of Preventive Cardiology*. 2023;30(18):2018-2031.

11.	Bao Y, Lee J, Thakur U, Ramkumar S, Marwick TH. Atrial fibrillation in cancer survivors–a systematic review and meta-analysis. *Cardio-Oncology*. 2023;9(1):29.

12.	Boyne DJ, Mickle AT, Brenner DR, et al. Long‐term risk of cardiovascular mortality in lymphoma survivors: A systematic review and meta‐analysis. *Cancer medicine*. 2018;7(9):4801-4813.

13.	Caruso V, Iacoviello L, Di Castelnuovo A, et al. Thrombotic complications in childhood acute lymphoblastic leukemia: a meta-analysis of 17 prospective studies comprising 1752 pediatric patients. *Blood*. 2006;108(7):2216-2222.

14.	Zhang F, Wang K, Du P, et al. Risk of stroke in cancer survivors: a meta-analysis of population-based cohort studies. *Neurology*. 2021;96(4):e513-e526.

15.	Balomenakis C, Papazoglou AS, Vlachopoulou D, et al. Risk of arterial thromboembolism, bleeding and mortality in atrial fibrillation patients with comorbid cancer: A systematic review and meta-analysis. *Hellenic Journal of Cardiology*. 2023;74:65-73.

16.	Wu Y-Y, Tang L, Wang M-H. Leukemia and risk of venous thromboembolism: a meta-analysis and systematic review of 144 studies comprising 162,126 patients. *Scientific Reports*. 2017;7(1):1167.

17.	Kostopoulos G, Doundoulakis I, Antza C, et al. Incident atrial fibrillation in patients with differentiated thyroid cancer: a meta-analysis. *Endocrine-related cancer*. 2021;28(5):325-335.

18.	Monaghan NP, Duckett KA, Nguyen SA, Newman JG, Albergotti WG, Kejner AE. Vascular events in patients with head and neck cancer: A systematic review and meta‐analysis. *Head & Neck*. 2024;46(7):1557-1572.

19.	Qian C, Yan H, Hu X, Zhang W, Liu H. Increased risk of venous thromboembolism in patients with brain tumors: a systematic review and meta-analysis. *Thrombosis Research*. 2016;137:58-63.

20.	Pastori D, Menichelli D, Di Rocco A, et al. Bleeding and thrombotic events in atrial fibrillation patients with cancer: a systematic review and meta-analysis. *Internal and Emergency Medicine*. 2023;18(2):655-665.

21.	Khan UT, Walker AJ, Baig S, Card TR, Kirwan CC, Grainge MJ. Venous thromboembolism and mortality in breast cancer: cohort study with systematic review and meta-analysis. *BMC cancer*. 2017;17:1-13.

22.	Wang D, Yu X, Sun Y, et al. Incidence of thrombosis at different sites during the follow-up period in essential thrombocythemia: a systematic review and meta-analysis. *Clinical and Applied Thrombosis/Hemostasis*. 2023;29:10760296231181117.

23.	Mauro E, Luca F, Tetta C, et al. Breast cancer and atrial fibrillation. *Journal of clinical medicine*. 2022;11(5):1417.

24.	Stone CR, Mickle AT, Boyne DJ, et al. Treatment for lymphoma and late cardiovascular disease risk: A systematic review and meta‐analysis. *Health science reports*. 2019;2(10):e135.

25.	Pergialiotis V, Vokotopoulou LV, Vlachos D-E, Liontos M, Kontomanolis E, Thomakos N. Pre-treatment thrombocytosis and ovarian cancer survival: A meta-analysis. *European Journal of Obstetrics & Gynecology and Reproductive Biology: X*. 2024:100312.

26.	Dongchen X, Tongyi L, Xueping M, Jingjing S, Quanhong L. Risk of mortality and other adverse outcomes from myocardial infarction in cancer survivors: a meta-analysis. *International Journal of Clinical Oncology*. 2023;28(1):41-51.

27.	Govsyeyev N, Malgor RD, Hoffman C, et al. A systematic review and meta-analysis of outcomes after acute limb ischemia in patients with cancer. *Journal of Vascular Surgery*. 2021;74(3):1033-1040. e1.

28.	Yang R, Wang H, Liu D, Li W. Incidence and risk factors of VTE in lung cancer: a meta-analysis. *Annals of Medicine*. 2024;56(1):2390200.

29.	Wang J, Yu J. A meta-analysis on the impact of concurrent or pre-existing cancer diagnosis on acute myocardial infarction outcomes. *PloS one*. 2025;20(1):e0318437.

30.	Hua X, Han S-H, Wei S-Z, Wu Y, Sha J, Zhu X-L. Clinical features of pulmonary embolism in patients with lung cancer: A meta-analysis. *PLoS One*. 2019;14(9):e0223230.

31.	Veiga VC, Peres SV, Ostolin TL, et al. Incidence of venous thromboembolism and bleeding in patients with malignant central nervous system neoplasm: Systematic review and meta-analysis. *Plos one*. 2024;19(6):e0304682.

32.	Roule V, Verdier L, Blanchart K, et al. Systematic review and meta-analysis of the prognostic impact of cancer among patients with acute coronary syndrome and/or percutaneous coronary intervention. *BMC Cardiovascular Disorders*. 2020;20:1-9.

33.	Betts MB, Liu X, Junqueira DR, et al. Risk of venous thromboembolism by cancer type: A network meta-analysis. Thieme Medical Publishers, Inc.; 2024:328-341.

34.	Harry J, Bucciol R, Finnigan D, Hashem H, Araki A, Othman M. The incidence of venous thromboembolism by type of solid cancer worldwide: A systematic review. *Cancer Epidemiology*. 2025;95:102764.
